# Quantifying the diagnostic time gap in precision oncology: A retrospective analysis of potential time savings from same-day cartridge-based molecular testing versus next-generation sequencing in cytology specimens

**DOI:** 10.1371/journal.pone.0360239

**Published:** 2026-10-16

**Authors:** Kevin Tran, Eveline Chen, W. Fraser Symmans, Gloria Hopkins Sura

**Affiliations:** 1 Department of Translational Molecular Pathology, Division of Pathology and Laboratory Medicine, The University of Texas MD Anderson Cancer Center, Houston, Texas, United States of America; 2 Sections of Cytopathology and Breast Pathology, Department of Anatomic Pathology, Division of Pathology and Laboratory Medicine, The University of Texas MD Anderson Cancer Center, Houston, Texas, United States of America; 3 Sections of Cytopathology and the Molecular Diagnostic Laboratory, Department of Anatomic Pathology, Division of Pathology and Laboratory Medicine, The University of Texas MD Anderson Cancer Center, Houston, Texas, United States of America; Teikyo University: Teikyo Daigaku, JAPAN

## Abstract

**Background:**

Next-generation sequencing (NGS) turnaround times (TAT) exceed two weeks and can delay the initiation of targeted therapy. Rapid cartridge-based platforms such as Idylla deliver single-gene or fusion results from minimal cytology material within hours, but time savings have not been systematically quantified.

**Methods:**

Forty-two cases of non-small cell lung carcinoma, thyroid carcinoma, melanoma, and mammary analogue secretory carcinoma of the salivary gland that underwent both Idylla testing (*KRAS*, *EGFR*, *BRAF,* and GeneFusion assays) and orthogonal NGS at a single academic center were reviewed. NGS TAT was calculated from specimen collection to report release, and Idylla results were modeled as available one day after collection. The primary outcome was per-case time savings (NGS TAT minus one day); treatment timelines were reconstructed from the medical record. Analyses were descriptive; no inferential testing was performed. A sensitivity analysis varied the modeled Idylla TAT from 1 to 3 days, and an adjusted estimate assigned no time saving to the two cartridge results that were not actionable on the day of collection.

**Results:**

The median NGS TAT across all 42 cases was 20.0 days (IQR, 17.0–23.8; range, 8–51). Against the modeled 1-day Idylla TAT, the median time savings was 19.0 days per case (IQR, 16.0–22.8; range, 7–50), totaling 858 patient-days. Assigning no time saving to those two cases gave a median of 18.0 days and 817 patient-days. No patient received therapy that was subsequently contradicted by the sequencing result. In thirteen patients, the sequencing interval alone exceeded three weeks, and in seven, no systemic therapy was underway while the result was awaited.

**Conclusions:**

Same-day cartridge-based testing from cytology and small biopsy specimens could provide actionable results a median of 19 days earlier than standard NGS. Whether this translates into earlier treatment initiation was not assessed and requires prospective evaluation.

## Introduction

Next-generation sequencing (NGS) has become central to the management of advanced and metastatic solid tumors, enabling the identification of therapeutically actionable genomic alterations across multiple tumor types [1,2]. These assays offer broad genomic coverage, but their workflows require multistep processing, including nucleic acid extraction, library preparation, sequencing, bioinformatic analysis, and expert interpretation, which typically results in turnaround times (TAT) that commonly exceed two weeks from specimen collection to finalized report [1–3]. This interval forces a choice between deferring treatment and beginning empiric chemotherapy or chemoimmunotherapy, and either course carries a cost for patients who prove to harbor an actionable driver. Deferral leaves symptomatic patients untreated; empiric treatment postpones targeted therapy and, where a checkpoint inhibitor has been given, can complicate the subsequent switch due to the risk of severe immune-related toxicity [4].

Rapid molecular testing platforms have emerged that complement comprehensive genomic profiling by detecting clinically actionable variants from minimal tissue input within hours. The Idylla platform (Biocartis Group NV, Mechelen, Belgium) is an automated, cartridge-based polymerase chain reaction (PCR) system that tests formalin-fixed paraffin-embedded (FFPE) tissue or cytology smear preparations with minimal sample preparation and a benchtop instrument runtime of 90–180 minutes, depending on the assay [3,5–7]. Prior analytical validation studies have demonstrated high concordance between Idylla assays and reference NGS methods across *KRAS*, *EGFR,* and *BRAF* mutation targets [3,6–8], and across gene fusions including *ALK*, *ROS1*, *RET*, *MET* exon 14 skipping, and *NTRK1/2/3* [3,5,7].

The platform is particularly suited to integration with cytopathology rapid on-site evaluation (ROSE), which makes reporting achievable within one day of collection. During ROSE, the aspirate smear is already prepared, and its adequacy and tumor cellularity are assessed by a cytopathologist at the time of the procedure, so testable material exists and has been qualified before the patient leaves the procedure suite. With the instrument sited adjacent to the ROSE area, specimens can be prepared, scraped, and loaded within minutes of collection; cartridges are run independently, instrument runtime is 90–180 minutes depending on the assay, and a report is generated automatically at the end of the run [3,6].

While the analytical performance of Idylla assays is well established, most published studies have addressed concordance and feasibility rather than quantifying the clinical time savings that integration of the assay into diagnostic workflows might achieve. This study addressed that gap. We retrospectively reconstructed real-world diagnostic timelines for patients with solid tumors who underwent standard-of-care NGS and compared them with a modeled scenario in which Idylla results were available within one day of collection, a value supported by the workflow described above, to quantify the per-case and aggregate time savings that same-day molecular testing could provide. An overview of the study design and clinical rationale is presented in Fig 1.

**Fig 1:**
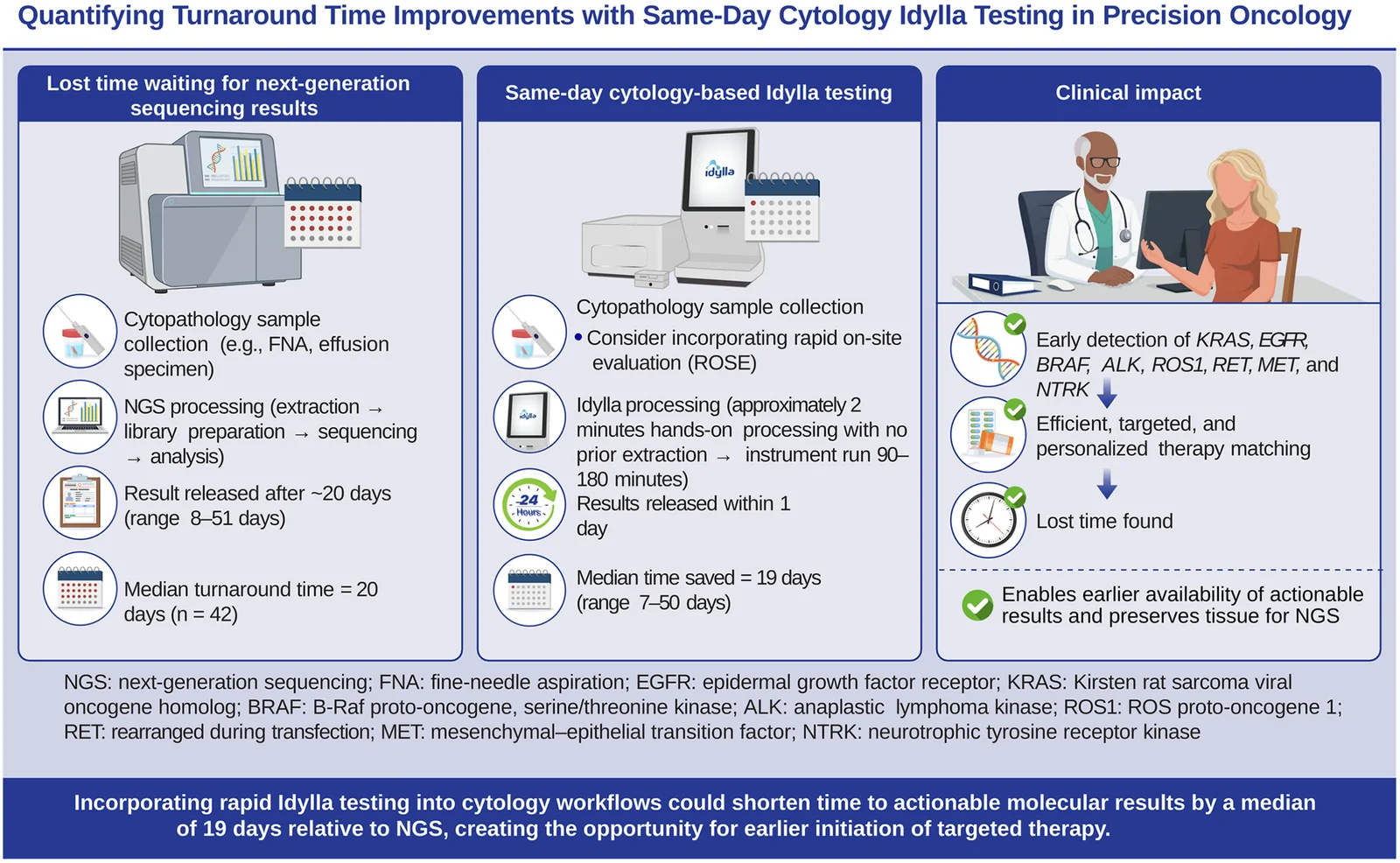
Graphical overview of the study design comparing standard next-generation sequencing and same-day Idylla cartridge-based PCR workflows. The left panel depicts the conventional NGS workflow from cytopathology sample collection through multistep processing to report release (median turnaround time, ~ 20 days; range, 8–51). The center panel illustrates the proposed Idylla-integrated workflow, in which cartridge-based PCR testing is performed with minimal hands-on time (approximately 2 minutes) and an instrument runtime of 90 to 180 minutes, depending on the assay, enabling results within one day, for a median modeled time saving of 19.0 days (range, 7–50) across the 42-case cohort. The right panel summarizes the clinical impact: rapid detection of actionable targets (*EGFR*, *KRAS*, *BRAF*, *ALK*, *ROS1*, *RET*, *MET*, and *NTRK*), earlier therapy matching, and earlier availability of actionable results while preserving tissue for comprehensive sequencing. Abbreviations: FNA, fine-needle aspiration; NGS, next-generation sequencing; PCR, polymerase chain reaction; ROSE, rapid on-site evaluation.

## Materials and methods

### Ethics statement and informed consent

This study was approved by the Institutional Review Board of The University of Texas MD Anderson Cancer Center (protocol number: 2023-0980) and was conducted in accordance with the principles of the Declaration of Helsinki. A waiver of informed consent was granted due to the minimal-risk, retrospective nature of the chart review. Clinical and pathological data were accessed for research purposes between November 30, 2023, and August 01, 2025. The corresponding author had access to information that could identify individual participants during data collection; all data were deidentified before being shared with the additional authors and used for analysis. All patient data were handled in accordance with HIPAA regulations.

### Study design and case selection

This was a retrospective time-interval analysis evaluating the potential reduction in molecular diagnostic TAT achievable by integrating same-day Idylla cartridge-based testing into cytopathology workflows. Forty-two clinical cases were drawn from a previously described analytical validation cohort in which clinical specimens underwent parallel testing by both Idylla (*KRAS*, *EGFR*, *BRAF*, and GeneFusion assays) and orthogonal in-house NGS at The University of Texas MD Anderson Cancer Center [3]. Orthogonal sequencing was performed as part of routine clinical care using one of four laboratory-developed assays, selected according to the class of alteration under assessment and which assay was in clinical service at the time of testing. Gene fusions and *MET* exon 14 skipping were assessed by the solid tumor genomic assay RNA fusions (STGA RNA), an RNA-based amplicon assay, and hotspot mutations in *KRAS*, *EGFR,* and *BRAF* by one of three DNA-based assays: the MD Anderson Mutation Analysis Precision Panel (MAPP), a hybridization capture assay; the solid tumor genomic assay DNA (STGA DNA), an amplicon assay that also serves as the reflex test when MAPP is unsuccessful; or the 50-gene cancer mutation scan (CMS50). Rearrangements were assessed by MAPP in one case; MAPP detects rearrangements from DNA across selected intronic regions rather than from RNA. Therefore, each cartridge assay was compared with the sequencing method used in routine clinical practice to report that class of alteration. Platform, chemistry, target coverage, specimen and cellularity requirements, limits of detection, and regulatory status for all four cartridge assays and all four sequencing assays are summarized in Tables 1 and S1–S4. Cartridge and sequencing assays were performed on material from the same diagnostic encounter but, in most cases, not on the same preparation. Orthogonal sequencing was most often performed on the FFPE cell block, whereas the cartridge assay used a destained aspirate smear obtained during the same aspiration procedure, so the two assays sampled the same specimen through different aliquots. In a small number of cases, the cartridge was run on an aspirate smear or a cell block section, while sequencing had been performed on a concurrently obtained core biopsy. No case was tested using both methods on the same slide. Performance characteristics of these assays in the parent validation cohort have been reported previously [3].

**Table 1:** Analytical characteristics of the Idylla cartridge assays and the orthogonal sequencing assays used as comparators.

Assay	Platform and chemistry	Alterations covered	Specimen, input, and cellularity requirements	Limit of detection	Turnaround and regulatory status
**Idylla cartridge assays**					
***KRAS* Mutation Assay**	Biocartis Idylla System; fully integrated cartridge-based real-time PCR from FFPE tissue. Internal control: *KRAS* Total	21 mutations in codons 12, 13, 59, 61, 117, and 146	FFPE sections, 5–10 µm; 50–600 mm² (5 µm) or 25–300 mm² (10 µm); ≥ 10% neoplastic cells	1% VAF observed in the parent validation study	120 min; research use only
***EGFR* Mutation Assay**	Biocartis Idylla System; fully integrated cartridge-based real-time PCR from FFPE tissue. Internal control: *EGFR* Total	Exon 18 (G719A/S/C), exon 19 deletions, exon 20 (T790M, S768I) and insertions, and exon 21 (L858R, L861Q)	1 × 5 µm FFPE section; > 10% neoplastic cells	0.9% VAF observed for p.T790M in the parent validation study	150 min; research use only
***BRAF* Mutation Assay**	Biocartis Idylla System; fully integrated cartridge-based real-time PCR from FFPE tissue. Internal control: RNaseP	Codon 600: V600E, V600E2, V600D, V600K, V600R, V600M, and wild type	FFPE sections, 5–10 µm; 50–600 mm² (5 µm) or 25–300 mm² (10 µm); > 50% neoplastic cells	1% VAF observed in the parent validation study	90 min; research use only
**GeneFusion Assay**	Biocartis Idylla System; cartridge-based reverse transcription and real-time PCR from FFPE tissue. Internal controls: *ERCC3*, *TMUB2*, wild-type *MET*, *KIF11*	17 *ALK*, 13 *ROS1* and 7 *RET* fusion variants; *MET* exon 14 skipping; expression imbalance for *ALK*, *ROS1*, *RET,* and *NTRK1/2/3*	1 × 5 µm FFPE section if area ≥20 mm², 3 × 5 µm if <20 mm²; ≥ 10% neoplastic cells	Not stated by the manufacturer	~180 min; research use only
**Orthogonal sequencing assays**					
**MAPP (2021–present). Comparator for hotspot mutations and for the rearrangement in one case**	Hybridization capture-based NGS on NovaSeq 6000 (Illumina); unique molecular index libraries; 2.1 Mb target enriched with custom 120-nucleotide probes	Sequence variants in 610 genes; copy number variants in 583 genes; rearrangements in 34 genes; microsatellite instability and tumor mutational burden	DNA from FFPE tissue or cytology specimens, including cell blocks and smears, with a paired normal specimen; minimum 50 ng genomic DNA; 20% neoplastic cells	5% VAF for sequence variants; 5 breakpoint molecules for fusions. Analytical sensitivity 99.2% (SNVs), 92.3% (indels), 89.1% (fusions); specificity approaching 100%	~10–14 calendar days after receipt in the laboratory; laboratory-developed test, CAP-accredited and CLIA-certified, not FDA cleared
**STGA DNA (2018–present). Comparator for hotspot mutations; also, the reflex assay when MAPP is unsuccessful**	Amplicon-based NGS on Ion GeneStudio S5 (Ion Torrent Systems)	Somatic mutations in 134 genes and copy number amplifications in 47 genes (146 total); point mutations, small indels, and gains only	DNA with matched non-tumor tissue for germline exclusion; amplicons adequately covered at ≥250 reads; 20% neoplastic cells	5–10% VAF for single nucleotide variants, depending on coverage depth; amplification reporting restricted to an estimated copy number ≥7	~10–14 calendar days after receipt in the laboratory; laboratory-developed test, CAP-accredited and CLIA-certified, not FDA cleared
**STGA RNA (2018–present). Comparator for gene fusions and *MET* exon 14 skipping**	Amplicon-based NGS on cDNA prepared from extracted RNA; alignment against a synthetic fusion genome with fusion calling by coverage analysis	Inter- and intragenic fusions in 51 genes, including *ALK*, *RET*, *ROS1*, *MET*, *NTRK1*, *NTRK2*, *NTRK3,* and NRG1	RNA; minimum 500,000 mapped cDNA target reads with mean sequence length ≥60 base pairs; a detected fusion must represent at least 1% of mapped reads; 20% neoplastic cells	No fixed limit of detection; sensitivity depends on fusion transcript expression and tumor purity. Requires both fusion partners and the junction to be flanked by amplicon primers	~10–14 calendar days after receipt in the laboratory; laboratory-developed test, CAP-accredited and CLIA-certified, not FDA cleared
**CMS50 (2013–2024). Comparator for hotspot mutations**	Ion AmpliSeq Cancer Hotspot Panel v2 on the Ion Torrent Personal Genome Machine; Ion 318 chips, up to 13 barcoded samples per run	Hotspots in 50 genes covering approximately 2,800 COSMIC mutations; silent mutations and common germline polymorphisms not reported	Genomic DNA at 20 ng/µL; amplicon read depth ≥250× for the tumor sample; 20% neoplastic cells	5–10% VAF for single-nucleotide variants, depending on coverage depth; variants called at ≥5% frequency with coverage ≥250×	~10–14 calendar days after receipt in the laboratory; laboratory-developed test, CAP-accredited and CLIA-certified, not FDA cleared

Neoplastic cellularity thresholds for the cartridge assays are pre-macrodissection requirements; specimens below the stated threshold are eligible after macrodissection or mapped scraping. Cellularity values recorded in S1–S4 Tables refer to the preparation loaded into the cartridge. All four sequencing assays require a minimum of 20% neoplastic cellularity, which is why no case in the present cohort falls below that level. Cartridge runtimes are instrument times from sample to result; sequencing turnaround is the laboratory interval from specimen receipt to report and is shorter than the observed collection-to-report intervals reported here, which also encompass accessioning, block preparation, and sign-out. Cartridges are run one specimen at a time and are not batched, whereas the amplicon-based sequencing assays multiplex barcoded specimens on a shared chip. Abbreviations: CAP, College of American Pathologists; cDNA, complementary DNA; CLIA, Clinical Laboratory Improvement Amendments; COSMIC, Catalogue of Somatic Mutations in Cancer; FDA, United States Food and Drug Administration; FFPE, formalin-fixed paraffin-embedded; NGS, next-generation sequencing; SNV, single-nucleotide variant; VAF, variant allele frequency.

The parent validation study assembled cell lines and residual archival cytology, small biopsy, and resection specimens from 110 patients with non-small cell lung carcinoma (n = 86), thyroid carcinoma (n = 14), melanoma (n = 9), and mammary analogue secretory carcinoma (MASC) of the salivary gland (n = 1) who had already undergone clinical sequencing, selecting specimens on the basis of residual material availability and deliberately including both variant-positive and wild-type cases so that assay accuracy could be assessed in both directions. From that cohort, we included every variant-positive case for which complete clinical and treatment data could be retrieved, irrespective of cartridge concordance or reportability. This cohort encompassed several tumor types: NSCLC (n = 35), thyroid carcinoma (n = 5; papillary, poorly differentiated, and anaplastic subtypes), melanoma (n = 1), and MASC of the salivary gland (n = 1). Wild-type cases and control cell line specimens were not eligible because a case without an actionable alteration has no targeted-therapy timeline against which a diagnostic interval can be assessed. The 42 cases represent 42 unique patients, each tested with a single cartridge assay type, so the assay-specific cohorts do not overlap. Several patients contributed more than one specimen preparation to the same assay run as a result of the parent validation study’s reproducibility assessments, yielding 56 Idylla specimen preparations across the 42 patients.

The prior validation study demonstrated sample-to-result times of 90–180 minutes, depending on the cartridge assay, using the Idylla platform (Table 1). Eight of 144 runs (5.6%) were non-informative, but these were not equivalent in origin: six were aspirate smears tested while still stained early in the parent validation study, before a destaining step was introduced; all six failed but were successful when repeated after destaining, and the remaining two reflected cartridge or instrument error. Among specimens prepared according to protocol, the non-reportable rate attributable to assay failure was therefore 2 of 138 runs (1.4%), leaving 136 of the 144 runs valid and available for concordance assessment. Among valid runs in the parent validation study, concordance with orthogonal methods was 100% for *KRAS* and *BRAF* assays, 94% for *EGFR* (33 of 35 runs), and 94% for GeneFusion (31 of 33 runs) [3].

Specimen types included FNA smears or cytospin preparations (Diff-Quik- or Papanicolaou-stained and subsequently destained) and FFPE cell block sections. Aspirate smears were decoverslipped by xylene soaking. Papanicolaou-stained smears were destained in 1% acid alcohol, and Diff-Quik-stained smears in methanol for 5–30 minutes, according to the time required to clear the stain from the individual slide. Tumor cellularity and mapping were assessed by a single board-certified cytopathologist (GHS) on the specific preparation subsequently loaded into the cartridge. Cellularity was recorded as the percentage of viable neoplastic cells within the mapped tumor area (S1–S4 Tables). Adequacy requirements are specified by the manufacturer for each cartridge type and vary between assays, from at least 10% neoplastic cells for the *KRAS*, *EGFR,* and GeneFusion cartridges to more than 50% for the *BRAF* cartridge (Table 1), alongside minimum and maximum tumor areas that depend on section thickness (25–300 mm² for aspirate smears and 10-micrometer sections, and 50–600 mm² for 5-micrometer sections). The lowest cellularity in the present cohort was 20%, which was a consequence of the sampling frame: eligibility required a prior successful orthogonal sequencing result, and the in-house sequencing assays require a minimum of 20% neoplastic cellularity, so no specimen below that level could have entered the cohort with sequencing data available for comparison. Clinical and pathological data, including diagnosis, stage, specimen type, tumor cellularity, orthogonal assay used, variant detected, and treatment history, were extracted from medical records and pathology reports.

### Timeline reconstruction and turnaround time calculation

For each case, the NGS TAT was defined as the number of calendar days between specimen collection and NGS report release. Treatment timelines were reconstructed through chart review, and the interval from specimen collection to initiation of first targeted or systemic therapy was recorded where applicable.

Each case was assigned an assumed Idylla TAT of one day in place of its observed NGS TAT. The value rests on a sequence of steps specific to cytology. During ROSE, a stained aspirate smear is prepared, and its adequacy and tumor cellularity are assessed by the cytopathologist at the time of the procedure, so no additional processing is required to generate testable material: the smear is decoverslipped and destained in 5–30 minutes, scraped with a scalpel blade, and loaded directly into the cartridge, which performs cell lysis and nucleic acid extraction internally. Hands-on time is approximately 2 minutes, cartridges are run independently, and instrument runtime is 90–180 minutes, depending on the assay [3]. A smear ready for testing before approximately 14:00 can therefore be reported the same day, and one ready can be reported the following morning. Cell block and small biopsy material reach the same endpoint by a different route: tissue processed on the day of the procedure yields a block the following morning, from which a section is scraped and loaded, giving a result on the first day after collection. One day is therefore an appropriate estimate for both preparation types. The contingencies that would extend either route, including delayed specimen receipt, weekend or holiday processing, and staffing constraints requiring batching, are not assumed in this calculation but are addressed in the sensitivity analysis described in the Statistical analysis section. The per-case time savings were calculated as:


$$\begin{array}{c} Time\;savings\;(days)\;=\;NGS\;TAT\;-\;\text{1} \end{array}$$

### Statistical analysis

Continuous variables were summarized as medians with interquartile ranges (IQRs) and full ranges. Descriptive statistics served as the primary analytical framework. Because the modeled Idylla TAT was assigned rather than observed and every observed NGS TAT exceeded it, the comparison is deterministic; no inferential hypothesis testing was performed. Treatment timelines were reconstructed from chart review to assess whether treatment decisions were made before or after NGS results became available and whether pre-NGS treatment choices were concordant with subsequent NGS findings. Analyses were stratified by assay type. Time to treatment was measured from specimen collection to initiation of the first systemic therapy rather than the first treatment of any modality because surgery and radiation are scheduled based on considerations largely independent of molecular results. A deterministic sensitivity analysis was performed in which the modeled Idylla TAT was varied across 1, 2, and 3 calendar days, covering same-day reporting from a destained smear, next-morning reporting from a cell block section, and the contingencies that would extend either route, including specimen receipt after the afternoon cut-off, weekend or holiday processing, and staffing constraints that require batching. For each case and each value, the time savings were recalculated as:


$$\begin{array}{c} Time\;savings\;(days)\;=\;NGS\;TAT\;-\;modeled\;Idylla\;TAT,\;where\;modeled\;Idylla\;TAT\;=\;\text{1},\;\text{2}\;or\;\text{3} \end{array}$$

A second scenario addressed cartridge results that would not have supported a treatment decision on the day of collection, so the patient reverted to the comprehensive sequencing timeline and the modeled saving was zero. Two such cases occurred in this cohort and are identified in the Results; each was assigned no time saving in an adjusted estimate reported alongside the primary figure. All calculations were performed using Python 3 with NumPy.

## Results

### Overall turnaround time analysis

Same-day cartridge-based testing was modeled to make actionable molecular results available a median of 19.0 days earlier than standard NGS across the 42-case cohort. Across all 42 evaluable cases, the median TAT for standard NGS reporting was 20.0 days (IQR, 17.0–23.8; range, 8–51) from specimen collection to report release (Table 2). Against the modeled Idylla TAT of one day, the median time savings was 19.0 days per case (IQR, 16.0–22.8; range, 7–50). The cumulative modeled saving across the 42-case cohort totaled 858 patient-days. In two cases (Cases 41 and 42), the cartridge result would not have supported a treatment decision on the day of collection: one was a false negative, and the other was an equivocal call requiring confirmatory testing. Assigning them no time savings gives a median of 18.0 days and a cumulative total of 817 patient-days, which we regard as the clinically realizable estimate. In the sensitivity analysis, raising the modeled Idylla TAT to 2 and 3 days gave median savings of 18.0 days (IQR, 15.0–21.8) and 17.0 days (IQR, 14.0–20.8), with cumulative savings of 816 and 774 patient-days. Because the shortest observed NGS TAT was 8 days, every case in which the cartridge returned an informative result retained at least 5 days of modeled time savings under the least favorable 3-day scenario, indicating that the direction and approximate magnitude of the finding do not depend on the specific value chosen. The upper tail of that distribution is where the interval mattered most: in 13 cases, the sequencing interval alone exceeded 3 weeks (Cases 5, 6, 8, 10, 12, 17, 18, 19, 32, 33, 35, 39, and 41), and in 7 of these (Cases 5, 6, 8, 18, 32, 33, and 39), no systemic therapy was underway while the result was awaited, so the wait was an untreated one rather than an interval during which treatment was already running.

**Table 2:** Comparison of NGS versus modeled Idylla turnaround times by assay type.

Biomarker/ assay	n	Median NGS TAT (days)	IQR (days)	Range (days)	Modeled Idylla TAT (days)	Median days saved (IQR)
*KRAS* Mutation Assay	10	20.5	17.5–24.2	14–39	1	19.5 (16.5–23.2)
*EGFR* Mutation Assay	8	20.0	18.0–29.5	17–51	1	19.0 (17.0–28.5)
*BRAF* Mutation Assay	11	19.0	17.5–21.0	10–24	1	18.0 (16.5–20.0)
GeneFusion Assay	13	20.0	15.0–27.0	8–35	1	19.0 (14.0–26.0)
**All cases**	**42**	**20.0**	**17.0–23.8**	**8–51**	**1**	**19.0 (16.0–22.8)**

Abbreviations: IQR, interquartile range; NGS, next-generation sequencing; TAT, turnaround time. The modeled Idylla TAT of 1 day represents a same-day cytopathology workflow integrating rapid on-site evaluation. Each case was tested with a single cartridge assay type; cases do not overlap between cohorts.

Fig 2 displays the per-case NGS TAT alongside the modeled 1-day cartridge-based PCR TAT, stratified by assay type.

**Fig 2:**
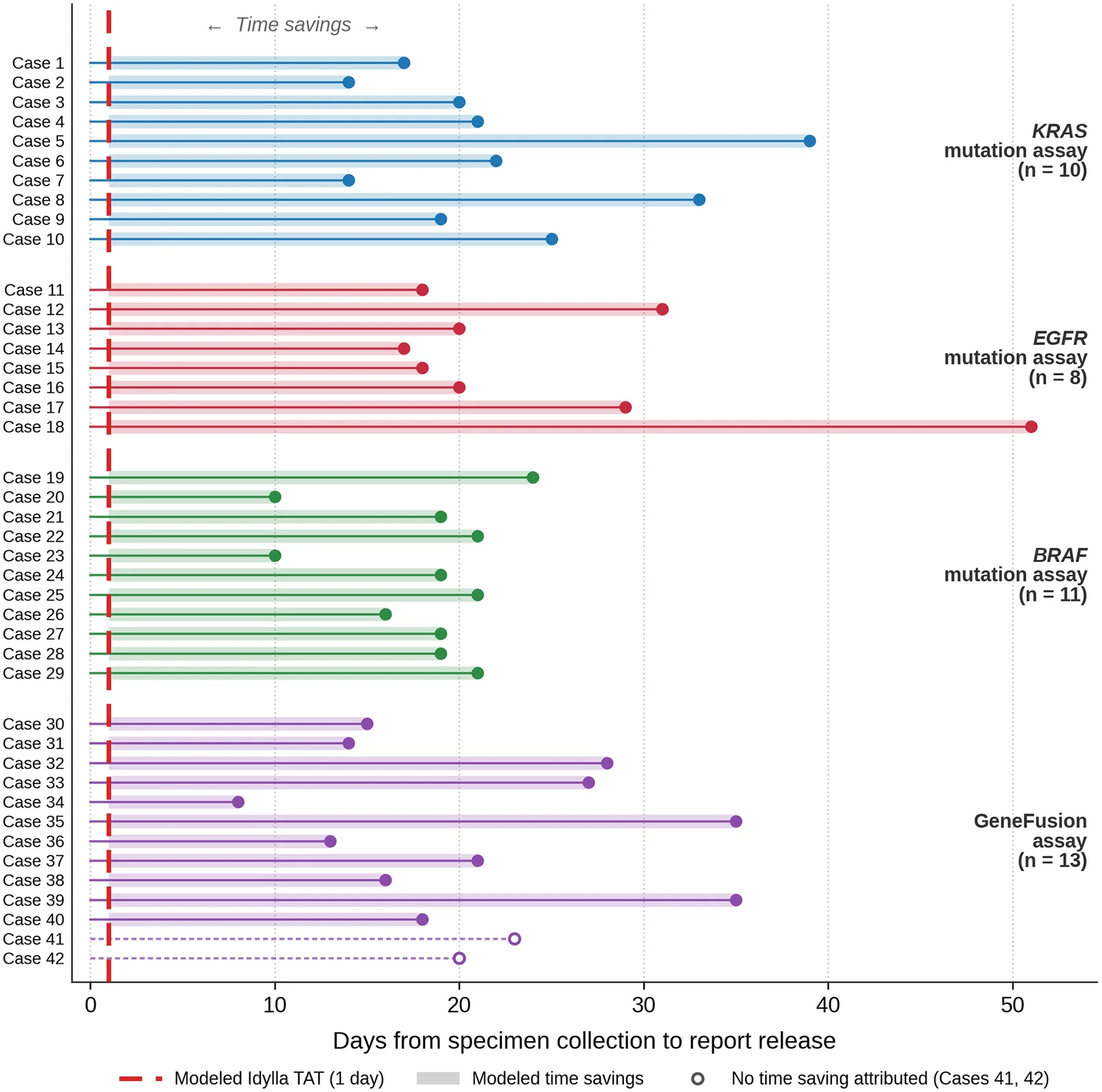
Per-case next-generation sequencing turnaround times and modeled time savings by assay type. Each bar represents the NGS TAT (days from specimen collection to report release) for one of the 42 cases, grouped by cartridge assay (*KRAS*, n = 10; *EGFR*, n = 8; *BRAF*, n = 11; GeneFusion, n = 13). The dashed line indicates the modeled 1-day Idylla TAT, and the shaded region between each bar and that line represents the modeled time savings for that case. Cases 41 and 42 are shown unshaded: in these cases, the cartridge returned a false-negative and an equivocal result, respectively, and would not have supported a treatment decision on the day of collection, so no time saving is attributed to them. The shaded regions sum to 817 patient-days, the clinically realizable estimate; including Cases 41 and 42 would give 858 patient-days.

Idylla results were concordant with orthogonal NGS for the actionable driver alteration in 40 of 42 cases. In two *EGFR* cases (Cases 17 and 18), the cartridge correctly identified the p.L858R driver, and p.T790M in Case 18 but additionally returned a spurious p.S768I call; in both cases, the quantification cycle and delta quantification cycle for the spurious call were well above those of the true positives. Both samples were repeated, and the assay software was updated from EGFR_RUO version 2.0 to 2.1; under the revised reporting cutoffs, neither call would have been auto-reported. The same artifact has been reported independently: in a prospective series of NSCLC samples, four of five cartridge-detected p.S768I calls in the initial analysis were not confirmed by sequencing, representing an 80% false-positive rate, and repeat testing on extracted DNA improved agreement with sequencing [9]. The two remaining cases were GeneFusion results that did not yield a definitive cartridge call and are described below. The cohort encompassed a range of specimen types (FNA smears, cytospin preparations, and FFPE cell block sections), with tumor cellularity ranging from 20% to 80%. Detailed specimen-level results, including input material, tumor cellularity, variants detected, and concordance status, are presented in S1–S4 Tables.

### Clinical context: Treatment timelines

Treatment timelines, provided in S1–S4 Tables, provide clinical context for interpreting the modeled time savings. Among patients harboring actionable driver mutations who had not yet started targeted therapy at the time of specimen collection, systemic therapy was initiated 8–106 days after collection, with the exception of one patient whose disease was localized at diagnosis and who began capmatinib only on progression to stage IV at 345 days (Case 1); three of them were managed with surgery or radiation first, at 118, 412, and 420 days. In several cases, the interval from collection to treatment exceeded the NGS TAT, reflecting additional delays attributable to insurance authorization [10], multidisciplinary review, care transitions, or clinical optimization before therapy.

To contextualize the potential impact of accelerated molecular results, treatment decisions were classified relative to the availability of in-house NGS results (Table 3). Three situations were distinguished. Six patients, five with NSCLC and one with anaplastic thyroid carcinoma (Cases 7, 11, 12, 17, 29 and 31), were already receiving target-directed therapy at the time of specimen collection, initiated on the basis of prior or outside molecular results; in these cases, the current specimen was obtained during ongoing treatment, and diagnostic turnaround was not on the critical path to therapy. Eleven patients, ten with NSCLC and one with melanoma (Cases 4, 9, 10, 19, 26, 27, 34, 35, 37, 41, and 42), began or continued empiric non-targeted therapy before the in-house report was available. In six of these, therapy had already begun before the current specimen was obtained, in each case before transfer to our institution; in the remaining five (Cases 10, 19, 26, 27, and 37), the regimen was selected during the diagnostic interval, and these are the patients in whom a same-day result could have informed the initial treatment decision. In one further case (Case 38, NSCLC), therapy began on the day the report was released.

**Table 3:** Classification of treatment decisions relative to in-house NGS result availability.

Case	Assay	Diagnosis & Stage	NGS TAT (days)	Treatment Initiation (days from collection)	Reason for Pre-NGS Treatment
Group A. Target-directed therapy already ongoing at specimen collection, initiated on the basis of prior or outside molecular results (n = 6)					
7	*KRAS*	NSCLC Stage IV	14	−493	Sotorasib initiated at an outside institution on prior molecular results; current specimen obtained during ongoing therapy
11	*EGFR*	NSCLC Stage IV	18	−565	Osimertinib initiated at an outside institution on prior molecular results
12	*EGFR*	NSCLC Stage IIB–IV	31	−191	Osimertinib initiated on prior molecular results, following cisplatin and pemetrexed at −324 days and surgery at −248 days
17	*EGFR*	NSCLC Stage IV	29	−427	Osimertinib and radiation initiated on *EGFR* status known from earlier sampling
29	*BRAF*	Anaplastic thyroid carcinoma Stage IV	21	−9	Dabrafenib and trametinib initiated on results received from an outside institution shortly before transfer
31	GeneFusion	NSCLC Stage IV	14	−227	Selpercatinib initiated at an outside institution on prior molecular results
Group B. Empiric non-targeted therapy begun or ongoing before the in-house NGS report (n = 11)					
4	*KRAS*	NSCLC Stage IV	21	−301	Carboplatin, pemetrexed, pembrolizumab, and radiation ongoing at collection; sotorasib followed the in-house report at 43 days
9	*KRAS*	NSCLC Stage IV	19	−1303	Carboplatin, pemetrexed, pembrolizumab, and radiation ongoing at collection from a prior disease episode
10	*KRAS*	NSCLC Stage IIIA–IV	25	14	Induction chemoimmunotherapy recommended but denied by the health plan; empiric cisplatin and docetaxel substituted before the report
19	*BRAF*	Melanoma Stage IV	24	17	Nivolumab and relatlimab begun 7 days before the *BRAF* result; first-line immunotherapy is guideline-concordant in melanoma irrespective of *BRAF* status
26	*BRAF*	NSCLC Stage IV	16	10	Carboplatin, pemetrexed, and pembrolizumab begun before the report; patient declined BRAF-directed therapy after the report
27	*BRAF*	NSCLC Stage IV	19	8	Carboplatin, pemetrexed, pembrolizumab, and radiation begun before the report; dabrafenib and trametinib followed at 24 days
34	GeneFusion	NSCLC Stage IV	8	−345	Carboplatin, pemetrexed, and radiation ongoing at collection; crizotinib followed the report at 11 days
35	GeneFusion	NSCLC Stage IV	35	−12	Pembrolizumab ongoing at collection; alectinib followed the report at 106 days
37	GeneFusion	NSCLC Stage IV	21	11	Carboplatin begun before the report; capmatinib followed at 77 days
41	GeneFusion	NSCLC Stage IV	23	−300	Carboplatin and pemetrexed ongoing at collection; the cartridge result was falsely negative, and alectinib followed the report at 93 days.
42	GeneFusion	NSCLC Stage IV	20	−120	Carboplatin and pemetrexed ongoing at collection and continued; the cartridge result was equivocal, and no targeted therapy was given.
Therapy initiated on the day the in-house NGS report was released (n = 1)					
38	GeneFusion	NSCLC Stage IV	16	16	Lorlatinib initiated on the same day the in-house report was released
Group C. Therapy followed the in-house NGS report (n = 24)					
Cases 1–3, 5, 6, 8, 13–16, 18, 20–25, 28, 30, 32, 33, 36, 39, and 40	Various	Various	10–51	Median 32.0 (IQR, 21.0–36.0), n = 17	Details of this group are given in the text.
Overall, no patient received therapy that was subsequently contradicted by the sequencing result.					

Treatment decisions for all 42 cases were classified into three groups according to their relationship to the in-house NGS report: target-directed therapy already ongoing at collection, empiric non-targeted therapy begun or ongoing before the report, and therapy following the report. For each pre-NGS case, the assay type, diagnosis, NGS turnaround time (TAT), treatment initiation relative to specimen collection, and the reason for pre-NGS treatment are provided. Negative values for treatment initiation indicate therapies begun before the current specimen collection, most often at an outside institution. Abbreviations: IQR, interquartile range; NGS, next-generation sequencing; NSCLC, non-small cell lung carcinoma; TAT, turnaround time.

Among the remaining 24 patients, therapy followed the report; 17 went on to receive systemic therapy at a median of 32.0 days from collection (IQR, 21.0–36.0; range, 14–345). The interval was defined as the time from specimen collection to initiation of the first systemic therapy, rather than to first treatment of any modality, because surgery and radiation are scheduled based on considerations largely independent of molecular results and would otherwise dominate the estimate. Seven patients did not receive systemic therapy after the report: two with NSCLC had treatment planned but never initiated, one having died before starting dabrafenib and trametinib (Case 21) and one having been lost to follow-up (Case 28); three were managed with surgery, radioactive iodine, or radiation alone, comprising two thyroid carcinomas and one salivary gland mammary analogue secretory carcinoma (Cases 22, 33, and 40); one with NSCLC received radiation and then declined further treatment (Case 3); and one with stage IA NSCLC underwent surgery with subsequent remission (Case 5). The upper end of the range reflects a single patient (Case 1) whose NSCLC was localized at diagnosis and who began capmatinib only on progression to stage IV at 345 days; excluding that case gives a median of 30.5 days (IQR, 20.8–35.2) across 16 patients. In this group, the median sequencing turnaround of 19.0 days accounted for much of the median 32.0 days to first systemic therapy, so molecular turnaround was a substantial but not exclusive component of the delay.

### *KRAS* mutation assay cohort

The Idylla *KRAS* Mutation assay was performed on 10 NSCLC cases spanning stages IA to IV, using FNA smears in nine cases and an FFPE cell block from pleural fluid in one, with tumor cellularity of 20–50% (S1 Table). In the two early-stage cases, local therapy came first: radiation at 18 days in Case 1 and surgery at 49 days in Case 5. Case 1 later progressed to stage IV and, with concurrent *MET* exon 14 skipping, began capmatinib at 345 days, with death at 735 days; Case 5 remained in remission at 1,350 days. Among the advanced cases, four began treatment 26–38 days after collection, and each regimen included radiation: radiation alone in Cases 3 and 8, and radiation with concurrent sotorasib or durvalumab in Cases 6 and 2. Case 3 subsequently declined further treatment. Three further patients (Cases 4, 7, and 9) were already receiving systemic therapy started at an outside institution 301, 493, and 1,303 days before the current specimen was obtained. Five patients harbored *KRAS* p.G12C (Cases 3, 4, 5, 6, and 7), of whom three received sotorasib (Cases 4, 6, and 7); the remaining two were managed with radiation or surgery. Among those with non-p.G12C alterations, four received platinum-based chemotherapy with or without immunotherapy (Cases 2, 8, 9, and 10). Case 10 began empiric cisplatin and docetaxel at 14 days, 11 days before the sequencing report, after induction chemoimmunotherapy was recommended but denied by the health plan; second-line pembrolizumab followed at 68 days.

### *EGFR* mutation assay cohort

Eight NSCLC cases were tested using the Idylla *EGFR* Mutation assay on FNA smears and one pleural fluid cytospin, with tumor cellularity of 25–60% (S2 Table). Patients harbored sensitizing *EGFR* mutations, including p.E746_A750del (exon 19 deletion), p.L858R, and p.L861Q, with one case (Case 18) also carrying the resistance mutation p.T790M. This cohort contained the two cases in which the cartridge returned a spurious p.S768I call (Cases 17 and 18) alongside the correctly identified driver described above; both were repeated, the reporting cutoffs subsequently revised, and neither call altered management, since osimertinib is indicated for the p.L858R and p.T790M variants that were correctly detected. Three patients were already receiving osimertinib when the specimen was obtained, having begun treatment 565, 427, and 191 days earlier (Cases 11, 17, and 12); Case 11 subsequently entered a trial of an *EGFR* mutant-selective inhibitor at 41 days. Among the four patients who began osimertinib after the report, with or without radiation, initiation occurred at 21–35 days (Cases 14, 16, 13, and 15), although Case 13 had undergone surgery and radiation for stage IA disease 1,541 days previously. Case 18 had the longest interval at 79 days, attributable to logistical difficulty in obtaining the prescription. At last contact, three patients remained on treatment at 964, 1,061, and 1,420 days (Cases 15, 12, and 13); four had died at 305, 432, 439, and 1,648 days (Cases 17, 16, 14, and 18), and one was continuing treatment at an outside hospital without further follow-up at 116 days (Case 11). Response was not formally assessed according to RECIST, and follow-up was to the last recorded contact. This cohort had the longest turnaround in the study, at 51 days in Case 18, and, within this cohort, the widest interval between report and treatment: that patient began osimertinib 28 days after the sequencing result became available.

### *BRAF* mutation assay cohort

Eleven cases (six NSCLC, four thyroid carcinomas, and one melanoma) were analyzed with the Idylla *BRAF* Mutation assay across FNA smears and FFPE materials, with tumor cellularity of 20–70% (S3 Table). Ten cases harbored *BRAF* p.V600E; the melanoma case (Case 19) harbored *BRAF* c.1798_1799delinsAA (p.V600K), and both variant classes were concordant with orthogonal NGS. The melanoma patient began nivolumab and relatlimab 17 days after collection and remained in remission at 933 days. Cartridge-based *BRAF* testing has been evaluated outside the lung setting: in a prospective series of 65 consecutive metastatic melanoma specimens, cartridge-based testing showed positive and negative predictive values of 100% for *BRAF* mutation detection, although the authors cautioned that the same cartridge missed three *NRAS* mutations and should not substitute for conventional molecular methods [11]. Comparable performance has been reported for thyroid fine-needle aspiration and tissue specimens [12,13], supporting extension of the same-day workflow to these tumor types. Among the six NSCLC cases, four initiated therapy 8–29 days after collection; in two cases (21 and 28), *BRAF*-directed therapy was planned but never started, one patient having died before initiation and one having been lost to follow-up. Dabrafenib and trametinib were given as first-line therapy in one case (20, at 14 days) and as second-line therapy in one case (27, at 24 days, following chemoimmunotherapy at 8 days). Two patients received platinum-based chemotherapy as their initial regimen (Cases 25 and 26), with pembrolizumab from the outset in Case 26 and durvalumab added at 106 days in Case 25. At last contact, one patient remained on active treatment at 835 days (Case 20) and two were under surveillance after partial remission at 903 and 1,834 days (Cases 27 and 25), while one had died at 154 days (Case 26). Among the thyroid carcinoma cases (n = 4), including papillary, poorly differentiated, and anaplastic subtypes, *BRAF*-directed treatment began at 17 days in one case (23, dabrafenib with radiation, with trametinib added at 82 days) and at 32 days in another (24, dabrafenib, trametinib, and radiation, followed by pembrolizumab at 643 days and everolimus at 787 days); treatment was already ongoing at the time of collection in one case (29, initiated 9 days before collection on the basis of outside molecular results), and in one papillary thyroid carcinoma (22), surgery with radioactive iodine followed at 420 days. At last contact, three patients were on active treatment or surveillance at 596, 644, and 754 days, and one had died at 926 days.

### GeneFusion assay cohort

Thirteen cases underwent Idylla GeneFusion testing on FNA smears and cell blocks (S4 Table), comprising eleven advanced NSCLC cases, one follicular variant papillary thyroid carcinoma, and one salivary gland mammary analogue secretory carcinoma (MASC). The two cases in which the cartridge did not return a definitive call both fell in this cohort, and they differed in kind. In Case 41, a stage IV lung adenocarcinoma, an *EML4::ALK*.E19A20 fusion reported by RNA-based sequencing was not detected by the cartridge, with no fusion-specific or expression-imbalance signal; the input contained 20% tumor over approximately 25 mm², meeting the assay adequacy thresholds of 10% and 25 mm². In Case 42, also a stage IV lung adenocarcinoma, the cartridge returned an equivocal call for *NTRK3,* whereas orthogonal RNA-based sequencing reported an *EML4::NTRK3* fusion. This result is more accurately described as technically concordant at the level of the gene involved, since the cartridge identified an *NTRK3* signal but below the threshold for a definitive call; it would have required confirmatory orthogonal *NTRK* testing before it could inform treatment. It was classified as non-concordant in the parent validation study, and the concordance figures cited above retain that classification. Because this cohort deliberately includes every discordant case from the parent validation study alongside only a subset of the concordant runs, the proportion of discordant cases within the cohort is a consequence of case selection and should not be interpreted as an estimate of assay accuracy. Among the eight patients who began targeted therapy after collection, initiation ranged from 11 to 106 days (Cases 34, 36, 38, 32, 39, 37, 41, and 35), with a median of 36.5 days. A ninth patient (Case 31) was already receiving selpercatinib at collection, and four (Cases 30, 33, 40, and 42) received no targeted agent. Five patients harbored *ALK* fusions: four received brigatinib, alectinib, or lorlatinib (Cases 32, 36, 38, and 35), and the fifth is Case 41, described above, who received alectinib at 93 days after the cartridge failed to detect the fusion. Those with ROS1 fusions were treated with crizotinib (Case 34) or repotrectinib (Case 39); the *MET* exon 14 skipping case that received a targeted agent (Case 37) was treated with capmatinib at 77 days, followed by an elzovantinib clinical trial at 221 days, while the second *MET* exon 14 case (Case 30) received carboplatin, pemetrexed, and radiation; a *RET* fusion–positive patient (Case 31) was treated with selpercatinib and subsequently enrolled in a clinical trial of a next-generation RET inhibitor. Treatment delays occurred in one case because of insurance approval (Case 37), while another patient (Case 38) received ALK-targeted therapy on the same day as the in-house NGS result release, having presented with prior external sequencing already completed. Among the eleven patients with NSCLC in this cohort, three remained on active treatment at last contact, at 994, 1,238, and 1,273 days after collection (Cases 30, 32, and 31, respectively). Seven had died by last contact, at 102, 117, 232, 240, and 453 days (Cases 39, 35, 42, 41, and 37), and at 842 days in two further cases (36 and 38); and one was lost to follow-up (Case 34). In Case 35, alectinib was started at 106 days, and the patient died 11 days later, illustrating that a molecular result can arrive too late to influence outcome even when it is acted upon. The single thyroid carcinoma in this cohort (Case 33, a follicular variant papillary thyroid carcinoma) received radiation at 118 days following remote thyroidectomy and was in partial remission on surveillance at 1,232 days. The single MASC patient (Case 40), harboring *ETV6::NTRK3*, underwent surgery and adjuvant radiation 412 days after collection and remains in remission under active surveillance.

## Discussion

This retrospective time-interval analysis demonstrated that integrating same-day Idylla cartridge-based molecular testing into cytopathology workflows could reduce molecular diagnostic TAT by a median of 19.0 days (IQR, 16.0–22.8) compared with standard NGS. Across 42 cases spanning four assay types, the median NGS TAT was 20.0 days versus a modeled cartridge TAT of one day. That figure is consistent with prior reports of extended NGS turnaround in clinical oncology, where reported medians range from approximately 14–26 days [1,3], including 19 days at an academic center using reflexive in-house sequencing [1]. It also extends a growing body of work showing that Idylla and comparable systems return single-gene or limited-panel results within hours at high concordance with comprehensive sequencing [3,5–8,14]. That rapid cartridge testing is faster than sequencing is not in itself a new observation; most prior publications have addressed analytical validation rather than downstream time savings, and the question addressed here is what that time is worth clinically.

Three features distinguish this analysis. First, earlier quantifications of time savings have addressed a single gene, most often *EGFR*, in a single tumor type [15–19]; here, the diagnostic interval is measured across four cartridge assay classes and four tumor types within one cohort, allowing the size of the gap to be compared between alteration classes. Second, we reconstructed case-level treatment timelines from the medical record rather than reporting turnaround alone, placing the diagnostic interval alongside the other intervals that precede therapy. Third, that reconstruction separates patients in whom a same-day result would have made the treatment decision sooner from those in whom it would have altered which treatment was chosen. For the 24 patients whose therapy followed the sequencing report (Table 3, Group C), the diagnostic interval sat directly on the path to treatment: a result available on the day of collection, rather than after a median of 19.5 days, could have brought forward the point at which a treatment decision became possible. For a smaller group, the benefit would have differed in kind rather than in degree. Five patients began empiric therapy between collection and the sequencing report (Cases 10, 19, 26, 27, and 37; a subset of Table 3, Group B), and in two of them an actionable driver was subsequently identified and treated (Cases 27 and 37), so a same-day result could have changed the initial regimen rather than only its timing. In no patient did the sequencing report contradict therapy already under way. The remaining 13 (Cases 4, 7, 9, 11, 12, 17, 29, 31, 34, 35, 38, 41, and 42, comprising all of Group A, the six Group B patients whose regimen predated collection, and Case 38) were already receiving systemic therapy when the specimen was obtained or began it on the day the sequencing report issued; for them, turnaround was not on the critical path. Separating these groups matters for assay deployment because it identifies both how much of the interval can be removed and for whom its removal would change the treatment decision rather than only its date. The contribution of this study is therefore not to demonstrate that rapid testing is faster, but to show how much of the diagnostic interval it removes and in which patients that removal would have mattered.

Shortening the diagnostic interval is one of several requirements for earlier treatment. The remaining barriers observed here, including prior authorization, prescription logistics, and transfers of care, are unaffected by how quickly the laboratory reports. Among the patients whose therapy followed the sequencing report, the median interval from collection to first systemic therapy was 32.0 days versus a median sequencing turnaround of 19.0 days, so molecular turnaround accounted for much of that interval but not all of it. In only two cases did the remainder exceed the sequencing turnaround (Cases 1 and 2). Long intervals between an available result and the start of targeted therapy nonetheless occurred across the cohort, irrespective of whether therapy followed the report: 28 days in Case 18, 56 days in Case 37, 70 days in Case 41, and 71 days in Case 35. These delays were attributed to difficulty obtaining the prescription in Case 18 and to insurance authorization in Case 37, and were not recorded in the other two cases. The opposite pattern also occurred: one patient died within a month of collection, only days after a sequencing report had consumed most of that interval, with targeted therapy planned but never started (Case 21).

Where molecular turnaround was the dominant interval, what that interval consists of determines how much of it a cartridge can remove. The 20.0-day median observed here is not a measure of assay duration. Sequencing turnaround in our laboratory is approximately 10–14 calendar days from receipt of the specimen to report release (Table 1), so approximately 6–10 days falls outside the assay itself, in accessioning, cell block preparation, transport between laboratories, and sign-out. The cartridge compresses both components rather than the analytical one alone: with ROSE, the specimen is already prepared and qualified at the time of the procedure, so the pre-analytical interval collapses to minutes and the analytical interval to 90–180 minutes, depending on the assay. For cell block material, a single overnight processing step remains, which is why one day rather than same-day reporting is the appropriate model. Batching adds a further delay that is invisible in assay runtime: sequencing runs are assembled from multiple specimens, so an individual case waits for a run to fill, whereas each cartridge is initiated as soon as the material is qualified. A third component is determined not by the laboratory at all but by when the test is ordered. In a series of 578 NSCLC biopsies, the mean interval from specimen to initiation of molecular testing was 30.0 weekdays for send-out testing and 27.4 weekdays when ordered in-house by the treating clinician, but 0.95 weekdays under a pathologist-initiated reflex protocol; reporting time was essentially unchanged across the three workflows at 11.1, 11.9, and 11.4 weekdays [20]. Reflex ordering therefore collapses the interval before the specimen reaches the molecular laboratory, and expert consensus supports pathologist-initiated testing for this reason [21].

Implementation in the cytopathology setting is supported by the platform’s operational characteristics [3,6,7]. The self-contained cartridge requires no nucleic acid extraction or library preparation and, in this cohort, ran on one or two destained aspirate smears or on one to three 10 µm sections from a cell block or core biopsy (Table 1). That minimal requirement preserves tissue for subsequent comprehensive sequencing, making the two approaches complementary rather than competing; when integrated with ROSE, it extends the on-site evaluation from morphologic adequacy assessment to same-day molecular triage [3,6].

Published series show that the interval between collection and treatment is shortened when rapid testing is introduced. In a Canadian series, adding rapid cartridge-based *EGFR* testing to the diagnostic pathway reduced the mean time to treatment by 10.1 days across the cohort and by 16.8 days, a 48% reduction, among patients treated on the basis of *EGFR* positivity [15]. A subsequent analysis from the same program reported a median turnaround of one day from laboratory receipt in routine practice [16]. A prospective bicentric study found that the interval from tumor sampling to EGFR-TKI initiation fell from 27.2 ± 8.3 to 11.4 ± 3.1 calendar days when the treatment decision followed the cartridge result rather than sequencing, although this analysis included only 22 patients [17]. In a multicenter European study of 1,474 paired analyses across 16 sites, 90% of cartridge results were returned within one week, compared with approximately 22 days for local reference methods [18]. The proportion of patients treated before sequencing returns are available is likewise not peculiar to our cohort: at a single academic center using a rapid *EGFR* polymerase chain reaction assay, 17 of 35 patients (49%) with *EGFR*-mutant disease began tyrosine kinase inhibitor therapy before sequencing results were available [19]. Delay in this interval is not without consequence: among 102 patients with *EGFR*-mutant NSCLC, 29 deteriorated rapidly before first-line tyrosine kinase inhibitor initiation, and 16 required hospitalizations, with deterioration in performance status associated with significantly worse prognosis [22]. None of these studies measured survival as a function of turnaround, so whether shortened diagnostic intervals improve outcomes remains an open question.

Guidelines recommend establishing histologic subtype and completing biomarker testing before systemic therapy is started; where an urgent start is required with biomarker results still pending, immunotherapy is held for one cycle and platinum-based chemotherapy is given alone so that subsequent targeted therapy is not compromised [23]. Waiting for molecular results therefore carries a therapeutic cost of its own, and a same-day result removes the need for that compromise in patients who prove to harbor an actionable driver: a negative cartridge result reinforces the decision to proceed with empiric therapy, whereas a positive result redirects planning toward targeted agents before the first cycle is given. That value is not universal. Some patients elect not to pursue systemic therapy or decline a specific agent once it is offered on the basis of personal goals, comorbidities, or logistical considerations.

Importantly, rapid single-gene and limited-panel assays do not replace comprehensive genomic profiling. The Idylla platform covers the most common therapeutically actionable mutations and fusions in NSCLC but does not encompass the full spectrum of guideline-recommended biomarkers, including *ERBB2* (HER2) mutations, uncommon *EGFR* variants, tumor mutational burden, microsatellite instability, and emerging or rare alterations [23,24]. Current guidelines recommend broad NGS panels as the standard approach for molecular characterization of advanced NSCLC [24]. Accordingly, rapid testing should be positioned as a front-line molecular triage tool that identifies common high-impact drivers within hours while comprehensive profiling proceeds in parallel.

A practical triage algorithm can be proposed from the parent validation study [3]. In NSCLC, cartridges may be run sequentially in order of population prevalence, beginning with *KRAS*, followed by *EGFR*, GeneFusion, and *BRAF*, and stopping once an alteration is identified, which yields a result in approximately 6–12 hours, depending on when the alteration is found; running the cartridges in parallel shortens this further at higher reagent cost. For thyroid carcinoma and melanoma, only the *BRAF* and GeneFusion cartridges are indicated, giving a processing time of approximately 2–6 hours. Cases in which all cartridges are negative should be routed to comprehensive sequencing, which additionally reports copy number variation, tumor mutational burden, microsatellite instability, and rare variants. We would apply rapid testing at initial diagnosis in symptomatic advanced disease, where tissue is limited, or where sequencing turnaround exceeds two weeks, but would not apply it to resistance profiling at progression, where the breadth of a comprehensive panel is required.

The setting in which this argument carries the greatest weight is the laboratory without on-site sequencing capacity, which must send specimens to a reference laboratory, and the resource-limited laboratory, where comprehensive sequencing may not be routinely available. For such centers, where send-out turnaround commonly exceeds three weeks, cartridge-based testing offers the largest absolute time saving of any setting, and the instrument requires neither a molecular technologist nor a bioinformatics pipeline. The economics are favorable at the level of the individual test: benchmarked against Centers for Medicare and Medicaid Services Clinical Laboratory Fee Schedule rates, which carried no phase-in payment reduction for calendar years 2025 or 2026, the cartridge assays used here cost approximately $386.50 for *KRAS*, $324.58 for *EGFR*, $175.40 for *BRAF,* and $597.91 for gene fusions, so that testing an NSCLC case with all four cartridges approaches $1,500, whereas thyroid and melanoma indications requiring only the *BRAF* and GeneFusion cartridges fall between approximately $200 and $800 [3]. Reported costs for comprehensive genomic profiling range from approximately $3,000 in list price to over $6,000 for commercial services [25,26]. These figures are not strictly comparable because the cartridge costs are Medicare reimbursement rates, whereas the profiling costs are list prices and commercial service charges; however, the difference in magnitude is large enough to support disease-specific triage rather than running all four assays on every case. Both sets of figures concern the cost of testing rather than the cost of waiting, which economic models have begun to quantify separately. A Canadian decision-tree analysis valued each week of delayed care at CAD 406, so that a strategy reaching targeted therapy at 9.2 rather than 5.1 weeks carried mean per-patient costs of CAD 5,632 against CAD 3,480 [27]. A United States payer model costed the delay and the treatment started before results were available as distinct components, at $3,228 and $5,991 per patient for sequential polymerase chain reaction testing versus $1,301 and $2,298 for upfront sequencing [28], extending an earlier decision-analytic model in which a shorter time to results was itself associated with savings for both public and commercial payers [29]. Those analyses compare sequential single-gene testing used in place of comprehensive sequencing rather than a rapid assay run alongside it, but they establish that the diagnostic interval carries a cost of its own. We did not perform a formal cost-effectiveness analysis, and these figures do not capture instrument acquisition, technologist time, or the cost of confirmatory sequencing that a negative cartridge result still requires. We would therefore attach three conditions to adoption in such settings: a defined reflex pathway to comprehensive sequencing when the rapid result is negative; recognition that the per-cartridge cost is favorable only when few assays are needed per patient; and acceptance that the restricted target coverage will miss a proportion of actionable alterations.

A same-day workflow is only as useful as the result it delivers, and the parent validation cohort allows the failure modes to be described concretely rather than as a global accuracy figure. Across 136 valid runs, the *KRAS* and *BRAF* assays were fully concordant with orthogonal sequencing. Two *EGFR* runs returned a spurious p.S768I call alongside a correctly identified p.L858R driver; in both, the quantification cycle and delta quantification cycle for the spurious call were well above those of the true positives, and the revision of reporting cutoffs between EGFR_RUO software versions 2.0 and 2.1 means that neither call would now be auto-reported. The two cartridge results that failed to deliver a definitive answer both occurred in the GeneFusion assay, and the distinction between them matters. In one, an *EML4::NTRK3* fusion was reported as equivocal: the assay identified *NTRK3* signal but below the threshold for a definitive call, so the result was technically concordant at the level of the gene involved yet could not have informed treatment without confirmatory orthogonal testing. In the other, an *EML4::ALK*.E19A20 fusion was not detected at all. The second is a true false negative and the more instructive of the two. That specimen contained 20% tumor over approximately 25 mm², close to the assay adequacy thresholds of 10% and 25 mm². The parent study did not establish a definitive cause for either discordance, noting only that the fusion may have been present at a level too low for detection. That patient subsequently received ALK-directed therapy; therefore, acting on the same-day negative result would have withheld a therapy that was subsequently indicated.

The comparison is also not a same-slide comparison. Because the cartridge and sequencing assays were run on different preparations of the same specimen, tumor content could differ between the two aliquots, and the cellularity and area recorded in S1–S4 Tables describe the material loaded into the cartridge rather than the material sequenced. Discordance in this setting may therefore reflect differences in sampling between preparations as well as assay analytical performance. The implication is not that rapid testing should be avoided, but that a negative cartridge result obtained near the adequacy floor cannot be treated as a negative finding and that comprehensive sequencing must proceed in parallel rather than be held pending the rapid result. It also argues for reporting tumor cellularity and specimen area alongside every rapid molecular result so that the clinician receiving a negative call can judge how much weight it should bear.

### Limitations

First, the one-day cartridge turnaround is modeled rather than observed. The supporting workflow is described above, but real-world turnaround varies with staffing, batching, and laboratory information system integration, so the reported savings are estimates under favorable conditions. Because that value is fixed and every observed NGS TAT exceeded it, the comparison is deterministic: no hypothesis test is meaningful, and the medians and interquartile ranges describe the magnitude of the modeled difference rather than test it. Prospectively measured cartridge turnaround times would permit a genuine paired comparison. Second, two of the 42 cartridge results would not have supported a same-day treatment decision. Both are retained because the calculation depends on sequencing turnaround rather than cartridge accuracy, and an adjusted estimate assigns them no saving. Runs yielding no result were confined to the parent cohort and bear on operational yield rather than turnaround.

Third, case selection was not consecutive: the parent cohort was assembled on residual specimen availability, and this analysis was further restricted to variant-positive cases with retrievable treatment timelines. The cohort is therefore enriched for actionable alterations and does not represent the unselected testing population, in which many cases return no actionable result; whether turnaround differs between variant-positive and wild-type cases was not assessed. The archival sampling frame has two further consequences. The orthogonal assays span platform generations in use from 2013 onward, which may itself contribute to the observed range of turnaround times. Cartridge performance below 20% neoplastic cellularity could not be assessed because such specimens have no comparator sequencing result, although these are precisely the specimens for which a rapid, low-input assay would be most valuable. Fourth, 42 cases limit precision and preclude subgroup analyses, and all data come from a single academic center. Fifth, the orthogonal comparator was not a single assay: MAPP in 18 cases, STGA RNA in 12, STGA DNA in seven, and CMS50 in five (S1–S4 Tables). Assay-specific turnaround therefore reflects both alteration class and sequencing platform, and differences between cohorts in Table 2 should not be attributed to the cartridge assay alone.

Sixth, cost-effectiveness was not assessed. Cartridges cost less per test than large panels, but the aggregate cost of up to four per patient and the reimbursement for rapid testing performed in parallel with sequencing both require economic evaluation [30]. The one published analysis of such a workflow found that 63% of 214 encounters were reimbursed, with payer variability of 0% to 100% [19]. Seventh, downstream outcomes were not measured as a function of turnaround. Prospective studies embedding rapid testing in real-time workflows are needed to establish whether the modeled savings translate into earlier treatment or improved outcomes. Finally, analytical error rates are established at CLIA validation and are independent of turnaround. Validation figures are given above; the false-positive rate is low, with the documented exception of the *EGFR* p.S768I artifact. Comprehensive sequencing remains the confirmatory assay, and a rapid preliminary result should be integrated within the full clinical and pathological context.

## Conclusions

This study modeled that same-day Idylla cartridge-based molecular testing from cytology and small biopsy specimens could make actionable molecular results available a median of 19 days earlier than standard NGS workflows. Used alongside comprehensive sequencing rather than in place of it, rapid front-line assays would narrow the diagnostic interval in cytopathology practice. Whether narrowing it changes treatment was not established here: in this cohort, the interval removed was more often one of waiting than one in which a treatment decision depended. Prospective studies are warranted to determine whether these time savings translate into earlier treatment initiation and improved patient outcomes.

## Supporting information

S1 Table Assay performance and modeled turnaround times for Idylla *KRAS* versus orthogonal NGS reporting. This table summarizes the analytical performance of the Idylla rapid cartridge-based assays and their modeled turnaround time compared with the corresponding orthogonal NGS results across all evaluated cases. For each case, calendar dates for NGS reporting and treatment initiation were converted to the number of days from specimen collection; negative values indicate events that occurred before collection (e.g., therapies begun at external institutions). The table also lists the diagnosis, disease stage at the time of orthogonal testing, summaries of treatment types, and the cytologic or small-biopsy input material used for each assay. Abbreviations: CMS50, 50-gene cancer mutation scan; DQ, Diff-Quik stain; F, female; FFPE, formalin-fixed, paraffin-embedded; FNA, fine-needle aspiration; M, male; MAPP, Mutation Analysis Precision Panel; MDACC, The University of Texas MD Anderson Cancer Center; NGS, next-generation sequencing; NSCLC, non-small cell lung carcinoma; Pap, Papanicolaou stain; STGA, solid tumor genomic assay.
(DOCX)

S2 Table Assay performance and modeled turnaround times for Idylla *EGFR* versus orthogonal NGS reporting. This table summarizes the analytical performance of the Idylla rapid cartridge-based assays and their modeled turnaround time compared with the corresponding orthogonal NGS results across all evaluated cases. For each case, calendar dates for NGS reporting and treatment initiation were converted to the number of days from specimen collection; negative values indicate events that occurred before collection (e.g., therapies begun at external institutions). The table also lists the diagnosis, disease stage at the time of orthogonal testing, summaries of treatment types, and the cytologic or small-biopsy input material used for each assay. *Spurious *EGFR* p.S768I resolved with software upgrade. Abbreviations: CMS50, 50-gene cancer mutation scan; DQ, Diff-Quik stain; F, female; FFPE, formalin-fixed, paraffin-embedded; FNA, fine-needle aspiration; M, male; MAPP, Mutation Analysis Precision Panel; MDACC, The University of Texas MD Anderson Cancer Center; NGS, next-generation sequencing; NSCLC, non-small cell lung carcinoma; Pap, Papanicolaou stain; STGA, solid tumor genomic assay.
(DOCX)

S3 Table Assay performance and modeled turnaround times for Idylla *BRAF* versus orthogonal NGS reporting. This table summarizes the analytical performance of the Idylla rapid cartridge-based assays and their modeled turnaround time compared with the corresponding orthogonal NGS results across all evaluated cases. For each case, calendar dates for NGS reporting and treatment initiation were converted to the number of days from specimen collection; negative values indicate events that occurred before collection (e.g., therapies begun at external institutions). The table also lists the diagnosis, disease stage at the time of orthogonal testing, summaries of treatment types, and the cytologic or small-biopsy input material used for each assay. Abbreviations: CMS50, 50-gene cancer mutation scan; DQ, Diff-Quik stain; F, female; FFPE, formalin-fixed, paraffin-embedded; FNA, fine-needle aspiration; M, male; MAPP, Mutation Analysis Precision Panel; MDACC, The University of Texas MD Anderson Cancer Center; NGS, next-generation sequencing; NSCLC, non-small cell lung carcinoma; Pap, Papanicolaou stain; STGA, solid tumor genomic assay.
(DOCX)

S4 Table Assay performance and modeled turnaround times for Idylla GeneFusion versus orthogonal NGS reporting. This table summarizes the analytical performance of the Idylla rapid cartridge-based assays and their modeled turnaround time compared with the corresponding orthogonal NGS results across all evaluated cases. For each case, calendar dates for NGS reporting and treatment initiation were converted to the number of days from specimen collection; negative values indicate events that occurred before collection (e.g., therapies begun at external institutions). The table also lists the diagnosis, disease stage at the time of orthogonal testing, summaries of treatment types, and the cytologic or small-biopsy input material used for each assay. Cases 41 and 42 are included although the cartridge did not return a definitive result: in Case 41, an *EML4::ALK*.E19A20 fusion was not detected, and in Case 42, an *NTRK3* call was equivocal and would have required confirmatory orthogonal *NTRK* testing. Both are retained because the primary outcome of this study, sequencing turnaround time, does not depend on cartridge performance. All cases from the parent validation study that were non-concordant or non-reportable and had complete clinical data are included here, whereas concordant cases represent a subset of those available; the proportion of non-concordant cases in this table therefore reflects case selection and is not an estimate of assay accuracy, for which the parent study denominators are given in the Materials and Methods. Abbreviations: DQ, Diff-Quik stain; F, female; FFPE, formalin-fixed, paraffin-embedded; FNA, fine-needle aspiration; M, male; MAPP, Mutation Analysis Precision Panel; MDACC, The University of Texas MD Anderson Cancer Center; NGS, next-generation sequencing; NSCLC, non-small cell lung carcinoma; Pap, Papanicolaou stain; STGA, solid tumor genomic assay.
(DOCX)

## References

[1] Vakkalagadda CV, Dressler DB, Sun Z, Fuchs J, Liu Y, Silberman P, et al. The impact of next-generation sequencing workflows on outcomes in advanced lung cancer: A retrospective analysis at one academic health system. Cancers (Basel). 2024;16(21):3654. doi: 10.3390/cancers16213654 39518093 PMC11545180

[2] Volders P-J, Aftimos P, Dedeurwaerdere F, Martens G, Canon J-L, Beniuga G, et al. A nationwide comprehensive genomic profiling and molecular tumor board platform for patients with advanced cancer. NPJ Precis Oncol. 2025;9(1):66. doi: 10.1038/s41698-025-00858-0 40065106 PMC11893761

[3] Iorgulescu JB, Yang RK, Roy-Chowdhuri S, Sura GH. Same-day molecular testing for targetable mutations in solid tumor cytopathology-The next frontier of the rapid on-site evaluation. Cancer Cytopathol. 2025;133(1):e22930. doi: 10.1002/cncy.22930 39746874

[4] Schoenfeld AJ, Arbour KC, Rizvi H, Iqbal AN, Gadgeel SM, Girshman J, et al. Severe immune-related adverse events are common with sequential PD-(L)1 blockade and osimertinib. Ann Oncol. 2019;30(5):839–44. doi: 10.1093/annonc/mdz077 30847464 PMC7360149

[5] Leone A, Muscarella LA, Graziano P, Tornese A, Grillo LR, Di Lorenzo A, et al. Robust performance of the novel research-use-only Idylla GeneFusion assay using a diverse set of pathological samples with a proposed 1-day workflow for advanced NSCLC evaluation. Cancers (Basel). 2022;15(1):292. doi: 10.3390/cancers15010292 36612287 PMC9818630

[6] De Luca C, Sgariglia R, Nacchio M, Pisapia P, Migliatico I, Clery E, et al. Rapid On-site Molecular Evaluation in thyroid cytopathology: A same-day cytological and molecular diagnosis. Diagn Cytopathol. 2020;48(4):300–7. doi: 10.1002/dc.24378 31904908

[7] Haragan A, Lee R. Rapid molecular profiling utilising minimal quantities of endobronchial ultrasound-guided aspirates for the detection of Epidermal Growth Factor Receptor, KRAS, ALK, ROS1, RET, NTRK and MET gene alterations from patients with non-small-cell lung carcinomas on the Biocartis Idylla™ platform. Cytopathology. 2024;35(5):648–53. doi: 10.1111/cyt.13406 38814131

[8] Pérez-Pérez M, de Sola-Llamas CG, Macías-García L. Diagnostic accuracy of the Idylla™ mutation test for detecting EGFR mutations in non-small cell lung cancer: A meta-analysis. Lung Cancer. 2025;205:108623. doi: 10.1016/j.lungcan.2025.108623 40554315

[9] Carnero-Gregorio M, Perera-Gordo E, de-la-Peña-Castro V, González-Martín JM, Delgado-Sánchez JJ, Rodríguez-Cerdeira C. High incidence of false positives in EGFR S768I mutation detection using the Idylla qPCR system in non-small cell lung cancer patients. Diagnostics. 2025;15(3):321. doi: 10.3390/diagnostics15030321 PMC11816987 39941251

[10] Murphy J, Beauchamp N, Sun KJ, Lau BD, Wilson RF, Lobner K, et al. Adverse effects of health plan prior authorization on clinical effectiveness and patient outcomes: A systematic review. Am J Med. 2026;139(1):24-32.e1. doi: 10.1016/j.amjmed.2025.08.018 40912445

[11] Vallée A, Denis-Musquer M, Herbreteau G, Théoleyre S, Bossard C, Denis MG. Prospective evaluation of two screening methods for molecular testing of metastatic melanoma: Diagnostic performance of BRAF V600E immunohistochemistry and of a NRAS-BRAF fully automated real-time PCR-based assay. PLoS One. 2019;14(8):e0221123. doi: 10.1371/journal.pone.0221123 31415669 PMC6695223

[12] Hamadou M, Lopez J, Benzerdjeb N, Cugnet-Anceau C, Schnoering G, Besançon J, et al. Feasibility and performance of the Idylla™ NRAS/BRAF cartridge mutation assay on thyroid liquid-based fine-needle aspiration. Diagn Cytopathol. 2021;49(12):1265–71. doi: 10.1002/dc.24897 34698448

[13] Yeo M-K, Jung M-K, Lee S-Y, Lee Y-M, Hur GM, Kim J-M. The usefulness of a novel fully automated PCR-based Idylla test for detection of the BRAF V600E mutation in thyroid tissue: Comparison with PNA-clamping PCR, real-time PCR and pyrosequencing. J Clin Pathol. 2017;70(3):260–5. doi: 10.1136/jclinpath-2016-204025 27543599

[14] Maher MH, Duose DY, Wistuba II, Luthra R, Arjuna S, Roy-Chowdhuri S, et al. A rapid turnaround time workflow for a cytological liquid biopsy assay using FNA supernatant specimens. Cancer Cytopathol. 2025;133(1):e22925. doi: 10.1002/cncy.22925 39704279

[15] Banyi N, Alex D, Hughesman C, McNeil K, N Ionescu D, Ma C, et al. Improving time-to-treatment for advanced non-small cell lung cancer patients through faster single gene EGFR testing using the Idylla™ EGFR testing platform. Curr Oncol. 2022;29(10):7900–11. doi: 10.3390/curroncol29100624 36290901 PMC9600153

[16] Fitzsimmons K, Hughesman C, Pataky R, Weymann D, D’Amours M-F, Alex D, et al. Ultrarapid EGFR testing in non-small cell lung carcinoma patients: Findings from a canadian clinical testing workflow. Lab Invest. 2025;105(12):104245. doi: 10.1016/j.labinv.2025.104245 41005709

[17] Petiteau C, Robinet-Zimmermann G, Riot A, Dorbeau M, Richard N, Blanc-Fournier C, et al. Contribution of the Idylla^TM^ system to improving the therapeutic care of patients with NSCLC through early screening of EGFR mutations. Curr Oncol. 2021;28(6):4432–45. doi: 10.3390/curroncol28060376 34898548 PMC8628756

[18] Behnke A, Cayre A, De Maglio G, Giannini G, Habran L, Tarsitano M, et al. FACILITATE: A real-world, multicenter, prospective study investigating the utility of a rapid, fully automated real-time PCR assay versus local reference methods for detecting epidermal growth factor receptor variants in NSCLC. Pathol Oncol Res. 2023;29:1610707. doi: 10.3389/pore.2023.1610707 36798672 PMC9927408

[19] Dagogo-Jack I, Azzolli CG, Fintelmann F, Mino-Kenudson M, Farago AF, Gainor JF, et al. Clinical utility of rapid EGFR genotyping in advanced lung cancer. JCO Precis Oncol. 2018;2018:PO.17.00299. doi: 10.1200/PO.17.00299 30370396 PMC6200882

[20] Smith BF, Hampel KJ, Sidiropoulos N. Benefits of implementing reflex genomic analysis for nonsmall cell lung cancer. J Appl Lab Med. 2024;9(1):28–40. doi: 10.1093/jalm/jfad104 38167774

[21] Gosney JR, Paz-Ares L, Jänne P, Kerr KM, Leighl NB, Lozano MD, et al. Pathologist-initiated reflex testing for biomarkers in non-small-cell lung cancer: Expert consensus on the rationale and considerations for implementation. ESMO Open. 2023;8(4):101587. doi: 10.1016/j.esmoop.2023.101587 37356358 PMC10485396

[22] Blanc-Durand F, Florescu M, Tehfe M, Routy B, Alameddine R, Tran-Thanh D, et al. Improvement of EGFR testing over the last decade and impact of delaying TKI initiation. Curr Oncol. 2021;28(2):1045–55. doi: 10.3390/curroncol28020102 33652831 PMC8025752

[23] Riely GJ, Wood DE, Aisner DL, Axtell AL, Bauman JR, Bharat A, et al. Non-small cell lung cancer, version 4.2026, NCCN clinical practice guidelines in oncology. J Natl Compr Canc Netw. 2026;24(4):e260017. doi: 10.6004/jnccn.2026.0017 41956107

[24] Lindeman NI, Cagle PT, Aisner DL, Arcila ME, Beasley MB, Bernicker EH, et al. Updated molecular testing guideline for the selection of lung cancer patients for treatment with targeted tyrosine kinase inhibitors: Guideline from the College of American Pathologists, the International Association for the Study of Lung Cancer, and the Association for Molecular Pathology. J Thorac Oncol. 2018;13(3):323–58. doi: 10.1016/j.jtho.2017.12.001

[25] Silas U, Blüher M, Bosworth Smith A, Saunders R. Fast in-house next-generation sequencing in the diagnosis of metastatic non-small cell lung cancer: A hospital budget impact analysis. J Health Econ Outcomes Res. 2023;10(1):111–8. doi: 10.36469/001c.77686 37389301 PMC10306161

[26] Johnston KM, Sheffield BS, Yip S, Lakzadeh P, Qian C, Nam J. Costs of in-house genomic profiling and implications for economic evaluation: A case example of non-small cell lung cancer (NSCLC). J Med Econ. 2020;23(10):1123–9. doi: 10.1080/13696998.2020.1789152 32597288

[27] Sheffield BS, Eaton K, Emond B, Lafeuille M-H, Hilts A, Lefebvre P, et al. Cost savings of expedited care with upfront next-generation sequencing testing versus single-gene testing among patients with metastatic non-small cell lung cancer based on current Canadian practices. Curr Oncol. 2023;30(2):2348–65. doi: 10.3390/curroncol30020180 36826141 PMC9955559

[28] Bestvina CM, Waters D, Morrison L, Emond B, Lafeuille M-H, Hilts A, et al. Cost of genetic testing, delayed care, and suboptimal treatment associated with polymerase chain reaction versus next-generation sequencing biomarker testing for genomic alterations in metastatic non-small cell lung cancer. J Med Econ. 2024;27(1):292–303. doi: 10.1080/13696998.2024.2314430 38391239

[29] Pennell NA, Mutebi A, Zhou Z-Y, Ricculli ML, Tang W, Wang H, et al. Economic impact of next-generation sequencing versus single-gene testing to detect genomic alterations in metastatic non-small-cell lung cancer using a decision analytic model. JCO Precis Oncol. 2019;3:1–9. doi: 10.1200/PO.18.00356 35100695

[30] Mirza M, Goerke L, Anderson A, Wilsdon T. Assessing the cost-effectiveness of next-generation sequencing as a biomarker testing approach in oncology and policy implications: A literature review. Value Health. 2024;27(9):1300–9. doi: 10.1016/j.jval.2024.04.023 38729563

