# Supplementary material for: Quantifying the diagnostic time gap in precision oncology: A retrospective analysis of potential time savings from same-day cartridge-based molecular testing versus next-generation sequencing in cytology specimens
Source: PLoS One. 2026 Oct 16;21(10):e0360239. doi: 10.1371/journal.pone.0360239 (PMC13674755; doi:10.1371/journal.pone.0360239)
Supplement: Supplementary file 1 [file pone.0360239.s001.docx]

**S1 Table. Assay performance and modeled turnaround times for Idylla *KRAS* versus orthogonal NGS reporting.** This table summarizes the analytical performance of the Idylla rapid cartridge-based assays and their modeled turnaround time compared with the corresponding orthogonal NGS results across all evaluated cases. For each case, calendar dates for NGS reporting and treatment initiation were converted to the number of days from specimen collection; negative values indicate events that occurred before collection (e.g., therapies begun at external institutions). The table also lists the diagnosis, disease stage at the time of orthogonal testing, summaries of treatment types, and the cytologic or small-biopsy input material used for each assay. ***Abbreviations:*** CMS50, 50-gene cancer mutation scan; DQ, Diff-Quik stain; F, female; FFPE, formalin-fixed, paraffin-embedded; FNA, fine-needle aspiration; M, male; MAPP, Mutation Analysis Precision Panel; MDACC, The University of Texas MD Anderson Cancer Center; NGS, next-generation sequencing; NSCLC, non-small cell lung carcinoma; Pap, Papanicolaou stain; STGA, solid tumor genomic assay.

| **Case / Diagnosis & Stage** | **NGS Result (Days from Collection)** | **Treatment Initiation (Days from Collection)** | **Idylla Sample** | **Tumor %; Specimen Area** | **Orthogonal NGS Result** | **Idylla Result** | **Orthogonal Assay** |
| --- | --- | --- | --- | --- | --- | --- | --- |
| **Case 1**  60, FFNA lung—NSCLC Stage IA3–IV | 17 days | Radiation to tumor confined to lung at Stage 1A3: 18 days  Capmatinib initiated after documentation stage IV disease: 345 days  Deceased: 735 days | Two aspirate smear slides, destained DQs | 25%; 100 mm² area | *KRAS* c.35G>C; p.G12A  *MET* exon 14 variant detected | Concordant | CMS50 |
| **Case 2**  65, MFNA lung—NSCLC Stage IV | 14 days | Durvalumab and radiation: 33 days  Carboplatin, pemetrexed, pembrolizumab: 642 days  Active therapy: 839 days | Two aspirate smear slides, destained Paps | 25%; 150 mm² area | *KRAS* c.35G>T; p.G12V | Concordant | MAPP |
| **Case 3**  83, FFNA lung—NSCLC Stage IIB–IV | 20 days | Radiation to primary tumor when at stage IIB and subsequent metastatic lesions: 34 days  Patient opted out of further treatment upon progression; hospice: 842 days | Two aspirate smear slides, destained DQs | 50%; 100 mm² area | *KRAS* c.34G>T; p.G12C | Concordant | MAPP |
|  |  |  | One aspirate smear slide, destained DQ | 50%; 150 mm² area | *KRAS* c.34G>T; p.G12C | Concordant | MAPP |
| **Case 4**  67, MFNA lung—NSCLC Stage IV | 21 days | Carboplatin, pemetrexed, pembrolizumab, and radiation (*initiated at an outside institution before transfer to MDACC*): –301 days prior to current specimen collection  Radiation: 16 days  Sotorasib: 43 days  Deceased: 304 days | One aspirate smear slide, destained DQ | 30%; 250 mm² area | *KRAS* c.34G>T; p.G12C | Concordant | MAPP |
|  |  |  | Two aspirate smear slides, destained DQ and Pap | 30%; 450 mm² area | *KRAS* c.34G>T; p.G12C | Concordant | MAPP |
| **Case 5**  66, FFNA lung—NSCLC Stage IA | 39 days | Surgery: 49 days  Remission: 1,350 days | One aspirate smear slide, destained DQ | 30%; 350 mm² area | *KRAS* c.34G>T; p.G12C | Concordant | STGA DNA |
| **Case 6**  69, FFNA 11L lymph node—NSCLC Stage IV | 22 days | Sotorasib and radiation: 26 days  Deceased: 648 days | One aspirate smear slide, destained DQ | 40%; 300 mm² area | *KRAS* c.34G>T; p.G12C | Concordant | STGA DNA |
| **Case 7**  74, MThoracentesis pleural fluid—NSCLC Stage IV | 14 days | Sotorasib (*initiated at an outside institution before transfer to MDACC*): –493 days prior to current sample collection  Deceased: 28 days | FFPE cell block, three 10 μm sections | 30%; 150 mm² area | *KRAS* c.34G>T; p.G12C | Concordant | MAPP |
| **Case 8**  68, MFNA 4R lymph node—NSCLC Stage III | 33 days | Radiation: 38 days  Carboplatin and Paclitaxel: 46 days  Durvalumab: 87 days  Active therapy: 971 days | Two aspirate smear slides, destained DQ and Pap | 40%; 100 mm² area | *KRAS* c.182A>T; p.Q61L | Concordant | MAPP |
| **Case 9**  58, FFNA station 7 lymph node—NSCLC Stage IV | 19 days | Carboplatin, pemetrexed, pembrolizumab, and radiation (*initiated at an outside institution before transfer to MDACC*): –1,303 days prior to current specimen collection  Deceased: 257 days | Two aspirate smear slides, destained DQ and Pap | 30%; 100 mm² area | *KRAS* c.38G>A; p.G13D | Concordant | MAPP |
|  |  |  | Two aspirate smear slides, destained DQ and Pap | 30%; 50 mm² area | *KRAS* c.38G>A; p.G13D | Concordant | MAPP |
| **Case 10**  59, F  FNA lung—NSCLC Stage IIIA–IV | 25 days | Cisplatin and docetaxel: 14 days  Pembrolizumab: 68 days  Deceased: 426 days | One aspirate smear slide, destained Pap | 20%; 25 mm² area | *KRAS* c.35G>T; p.G12V | Concordant | STGA DNA |
|  |  |  | One aspirate smear slide, destained Pap | 20%; 100 mm² area | *KRAS* c.35G>T; p.G12V | Concordant | STGA DNA |
