# Supplementary material for: Quantifying the diagnostic time gap in precision oncology: A retrospective analysis of potential time savings from same-day cartridge-based molecular testing versus next-generation sequencing in cytology specimens
Source: PLoS One. 2026 Oct 16;21(10):e0360239. doi: 10.1371/journal.pone.0360239 (PMC13674755; doi:10.1371/journal.pone.0360239)
Supplement: Supplementary file 2 [file pone.0360239.s002.docx]

**S2 Table. Assay performance and modeled turnaround times for Idylla *EGFR* versus orthogonal NGS reporting.** This table summarizes the analytical performance of the Idylla rapid cartridge-based assays and their modeled turnaround time compared with the corresponding orthogonal NGS results across all evaluated cases. For each case, calendar dates for NGS reporting and treatment initiation were converted to the number of days from specimen collection; negative values indicate events that occurred before collection (e.g., therapies begun at external institutions). The table also lists the diagnosis, disease stage at the time of orthogonal testing, summaries of treatment types, and the cytologic or small-biopsy input material used for each assay. *Spurious *EGFR* p.S768I resolved with software upgrade. ***Abbreviations:*** CMS50, 50-gene cancer mutation scan; DQ, Diff-Quik stain; F, female; FFPE, formalin-fixed, paraffin-embedded; FNA, fine-needle aspiration; M, male; MAPP, Mutation Analysis Precision Panel; MDACC, The University of Texas MD Anderson Cancer Center; NGS, next-generation sequencing; NSCLC, non-small cell lung carcinoma; Pap, Papanicolaou stain; STGA, solid tumor genomic assay.

| **Case / Diagnosis & Stage** | **NGS Result (Days from Collection)** | **Treatment Initiation (Days from Collection)** | **Idylla Sample** | **Tumor %; Specimen Area** | **Orthogonal Result** | **Idylla Result** | **Orthogonal Assay** |
| --- | --- | --- | --- | --- | --- | --- | --- |
| **Case 11**  49, MThoracentesis, pleural fluid—NSCLC Stage IV | 18 days | Osimertinib (*initiated at an outside institution before transfer to MDACC*): –565 days prior to current sample collection  Enrolled in clinical trial involving an *EGFR* mutant-selective inhibitor: 41 days  Continuing treatment at outside hospital; no follow-up: 116 days | One cytospin slide, destained Pap | 30%; 200 mm² area | *EGFR* c.2235_2249del; p.E746_A750del (exon 19 del) | Concordant | MAPP |
| **Case 12**  73, FFNA lung—NSCLC Stage IIB–IV | 31 days | Cisplatin and pemetrexed (*initiated at an outside institution before transfer to MDACC*): –324 days prior to current sample collection  Surgery (*initiated at an outside institution before transfer to MDACC*): –248 days prior to current sample collection  Osimertinib: –191 days prior to current sample collection  Active treatment: 1,061 days | One aspirate smear slide, destained Pap | 60%; 150 mm² area | *EGFR* c.2573T>G; p.L858R | Concordant | CMS50 |
|  |  |  | One aspirate smear slide, destained DQ | 60%; 250 mm² area | *EGFR* c.2573T>G; p.L858R | Concordant | CMS50 |
| **Case 13**  35, FFNA lung—NSCLC Stage IA–IIB | 20 days | Surgery and radiation at stage 1A disease: –1,541 days prior to current sample collection  Osimertinib and radiation: 33 days  Active treatment: 1,420 days | Two aspirate smear slides, destained DQ | 25%; 100 mm² area | *EGFR* c.2582T>A; p.L861Q | Concordant | STGA DNA |
| **Case 14**  78, MFNA lung—NSCLC Stage IV | 17 days | Osimertinib and radiation: 21 days  Deceased: 439 days | One aspirate smear slide, destained DQ | 30%; 300 mm² area | *EGFR* c.2582T>A; p.L861Q | Concordant | STGA DNA |
|  |  |  | One aspirate smear slide, destained DQ | 30%; 350 mm² area | *EGFR* c.2582T>A; p.L861Q | Concordant | STGA DNA |
| **Case 15**  68, FFNA lung—NSCLC Stage IV | 18 days | Osimertinib and radiation: 35 days  Active treatment: 964 days | One aspirate smear slide, destained DQ | 40%; 100 mm² area | *EGFR* c.2236_2250del; p.E746_A750del (exon 19 del) | Concordant | MAPP |
| **Case 16**  72, MFNA lung—NSCLC Stage IV | 20 days | Osimertinib; radiation: 26 days  Deceased: 432 days | One aspirate smear slide, destained DQ | 25%; 100 mm² area | *EGFR* c.2582T>A; p.L861Q | Concordant | STGA DNA |
| **Case 17**  69, FFNA lung—NSCLC Stage IV | 29 days | Osimertinib and radiation (*EGFR mutation status was known* *from prior sampling*): –427 days prior to current sample collection  Deceased: 305 days | One aspirate smear slide, destained Pap | 50%; 300 mm² area | *EGFR* c.2573T>G; p.L858R | Concordant * | MAPP |
| **Case 18**  68, FFNA lung—NSCLC Stage IV | 51 days | Osimertinib and radiation: 79 days  Pemetrexed and radiation: 1,297 days  Deceased: 1,648 days | One aspirate smear slide, destained DQ | 50%; 200 mm² area | *EGFR* c.2573T>G; p.L858R &  *EGFR* c.2369T>C; p.T790M | Concordant * | MAPP |
