# Supplementary material for: Quantifying the diagnostic time gap in precision oncology: A retrospective analysis of potential time savings from same-day cartridge-based molecular testing versus next-generation sequencing in cytology specimens
Source: PLoS One. 2026 Oct 16;21(10):e0360239. doi: 10.1371/journal.pone.0360239 (PMC13674755; doi:10.1371/journal.pone.0360239)
Supplement: Supplementary file 3 [file pone.0360239.s003.docx]

**S3 Table. Assay performance and modeled turnaround times for Idylla *BRAF* versus orthogonal NGS reporting.** This table summarizes the analytical performance of the Idylla rapid cartridge-based assays and their modeled turnaround time compared with the corresponding orthogonal NGS results across all evaluated cases. For each case, calendar dates for NGS reporting and treatment initiation were converted to the number of days from specimen collection; negative values indicate events that occurred before collection (e.g., therapies begun at external institutions). The table also lists the diagnosis, disease stage at the time of orthogonal testing, summaries of treatment types, and the cytologic or small-biopsy input material used for each assay. ***Abbreviations:*** CMS50, 50-gene cancer mutation scan; DQ, Diff-Quik stain; F, female; FFPE, formalin-fixed, paraffin-embedded; FNA, fine-needle aspiration; M, male; MAPP, Mutation Analysis Precision Panel; MDACC, The University of Texas MD Anderson Cancer Center; NGS, next-generation sequencing; NSCLC, non-small cell lung carcinoma; Pap, Papanicolaou stain; STGA, solid tumor genomic assay.

| **Case / Diagnosis & Stage** | **NGS Result (Days from Collection)** | **Treatment Initiation (Days from Collection)** | **Idylla Sample** | **Tumor %; Specimen Area** | **Orthogonal Result** | **Idylla Result** | **Orthogonal Assay** |
| --- | --- | --- | --- | --- | --- | --- | --- |
| **Case 19**  72, MFNA lung—Melanoma Stage IV | 24 days | Nivolumab and relatlimab: 17 days  In remission: 933 days | One aspirate smear slide, destained Pap | 20%; 300 mm² | *BRAF* c.1798_1799delinsAA; p.V600K | Concordant | CMS50 |
|  |  |  | Two aspirate smear slides, destained Pap and DQ | 20%; 250 mm² | *BRAF* c.1798_1799delinsAA; p.V600K | Concordant | CMS50 |
| **Case 20**  65, MFNA station 7 lymph node—NSCLC Stage IV | 10 days | Dabrafenib and trametinib: 14 days  Active treatment: 835 days | One aspirate smear slide, destained Pap | 30%; 450 mm² area | *BRAF* c.1799T>A; p.V600E | Concordant | MAPP |
|  |  |  | One aspirate smear slide, destained DQ | 30%; 450 mm² area | *BRAF* c.1799T>A; p.V600E | Concordant | MAPP |
| **Case 21**  79, MFNA 4R lymph node—NSCLC Stage IV | 19 days | Dabrafenib and trametinib treatment planned; patient passed before initiation: 27 days | One aspirate smear slide, destained DQ | 50%; 200 mm² area | *BRAF* c.1799T>A; p.V600E | Concordant | CMS50 |
| **Case 22**  57, F FNA right neck lymph node—papillary thyroid carcinoma Stage II | 21 days | Surgery and subsequent radioactive iodine therapy: 420 days  Active surveillance: 754 days | FFPE core biopsy block, one 10 μm section | 30%; 50 mm² | *BRAF* c.1799T>A; p.V600E | Concordant | MAPP |
|  |  |  | FFPE resection block, 10 μm section | 80%; 500 mm² | *BRAF* c.1799T>A; p.V600E | Concordant | MAPP |
| **Case 23**  49, FFNA lung—poorly differentiated thyroid carcinoma Stage IV | 10 days | Dabrafenib and radiation: 17 days  Dabrafenib and trametinib: 82 days  Active treatment: 596 days | FFPE core biopsy block, two 10 μm sections | 40%; 50 mm² area | *BRAF* c.1799T>A; p.V600E | Concordant | MAPP |
| **Case 24**  72, F Core biopsy, neck mass—anaplastic thyroid carcinoma Stage IV | 19 days | Dabrafenib, trametinib, and radiation: 32 days  Pembrolizumab: 643 days  Everolimus: 787 days  Deceased: 926 days | FFPE core biopsy block, two 10 μm sections | 20%; 50 mm² area | *BRAF* c.1799T>A; p.V600E | Concordant | MAPP |
| **Case 25**  72, FFNA supraclavicular lymph node—NSCLC Stage IIIB | 21 days | Carboplatin, paclitaxel, and radiation: 29 days  Carboplatin, paclitaxel, radiation, and durvalumab: 106 days  Partial remission achieved; on active surveillance: 1,834 days | One aspirate smear slide, destained DQ | 30%; 150 mm² area | *BRAF* c.1799T>A; p.V600E | Concordant | STGA DNA |
| **Case 26**  82, M FNA lung—NSCLC Stage IV | 16 days | Carboplatin, pemetrexed, and pembrolizumab: 10 days  Deceased: 154 days | One aspirate smear slide, destained DQ | 40%; 250 mm² area | *BRAF* c.1799T>A; p.V600E | Concordant | MAPP |
| **Case 27**  68, FFNA supraclavicular lymph node—NSCLC Stage IV | 19 days | Carboplatin, pemetrexed, pembrolizumab, and radiation: 8 days  Dabrafenib and trametinib: 24 days  Pembrolizumab: 134 days  Partial remission; on active surveillance: 903 days | One aspirate smear slide, destained Pap | 30%; 300 mm² area | *BRAF* c.1799T>A; p.V600E | Concordant | MAPP |
| **Case 28**  73, M  FNA lung—NSCLC Stage IV | 19 days | Dabrafenib, trametinib, and pembrolizumab plan created, but patient lost to follow-up: 27 days | One aspirate smear slide, destained Pap | 50%; 300 mm² area | *BRAF* c.1799T>A; p.V600E | Concordant | CMS50 |
| **Case 29**  67, M FNA thyroid—anaplastic thyroid carcinoma Stage IV | 21 days | Dabrafenib and trametinib (*initiated based on results received from an outside institution before transfer to MDACC*): –9 days prior to the current sample collection  Pembrolizumab: 36 days  Active treatment: 644 days | FFPE cell block, two 10 μm sections | 70%; 100 mm² | *BRAF* c.1799T>A; p.V600E | Concordant | MAPP |
|  |  |  | FFPE cell block, one 10 μm section | 70%; 50 mm² | *BRAF* c.1799T>A; p.V600E | Concordant | MAPP |
