# Supplementary material for: Quantifying the diagnostic time gap in precision oncology: A retrospective analysis of potential time savings from same-day cartridge-based molecular testing versus next-generation sequencing in cytology specimens
Source: PLoS One. 2026 Oct 16;21(10):e0360239. doi: 10.1371/journal.pone.0360239 (PMC13674755; doi:10.1371/journal.pone.0360239)
Supplement: Supplementary file 4 [file pone.0360239.s004.docx]

**S4 Table. Assay performance and modeled turnaround times for Idylla GeneFusion versus orthogonal NGS reporting.** This table summarizes the analytical performance of the Idylla rapid cartridge-based assays and their modeled turnaround time compared with the corresponding orthogonal NGS results across all evaluated cases. For each case, calendar dates for NGS reporting and treatment initiation were converted to the number of days from specimen collection; negative values indicate events that occurred before collection (e.g., therapies begun at external institutions). The table also lists the diagnosis, disease stage at the time of orthogonal testing, summaries of treatment types, and the cytologic or small-biopsy input material used for each assay. Cases 41 and 42 are included although the cartridge did not return a definitive result: in Case 41, an *EML4::ALK*.E19A20 fusion was not detected, and in Case 42, an *NTRK3* call was equivocal and would have required confirmatory orthogonal *NTRK* testing. Both are retained because the primary outcome of this study, sequencing turnaround time, does not depend on cartridge performance. All cases from the parent validation study that were non-concordant or non-reportable and had complete clinical data are included here, whereas concordant cases represent a subset of those available; the proportion of non-concordant cases in this table therefore reflects case selection and is not an estimate of assay accuracy, for which the parent study denominators are given in the Materials and Methods. ***Abbreviations:*** DQ, Diff-Quik stain; F, female; FFPE, formalin-fixed, paraffin-embedded; FNA, fine-needle aspiration; M, male; MAPP, Mutation Analysis Precision Panel; MDACC, The University of Texas MD Anderson Cancer Center; NGS, next-generation sequencing; NSCLC, non-small cell lung carcinoma; Pap, Papanicolaou stain; STGA, solid tumor genomic assay.

| **Case / Diagnosis & Stage** | **NGS Result (Days from Collection)** | **Treatment Initiation (Days from Collection)** | **Idylla Sample** | **Tumor %; Specimen Area** | **Orthogonal Result** | **Idylla Result** | **Orthogonal Assay** |
| --- | --- | --- | --- | --- | --- | --- | --- |
| **Case 30**  75, FFNA lung—NSCLC Stage IV | 15 days | Carboplatin, pemetrexed, and radiation: 20 days  Active treatment: 994 days | Two aspirate smear slides, destained Pap and DQ | 25%; 50 mm² | *MET::MET*.M13M15 | Concordant | STGA RNA |
|  |  |  | One aspirate smear slide, destained DQ | 30%; 200 mm² | *MET::MET*.M13M15 | Concordant | STGA RNA |
| **Case 31**  74, FFNA axillary lymph node—NSCLC Stage IV | 14 days | Selpercatinib (*initiated at an outside institution before transfer to MDACC):* –227 days prior to current sample collection  Carboplatin, pemetrexed, and radiation: 217 days  Enrolled in clinical trial involving a next-generation selective RET inhibitor: 1,051 days  Active treatment: 1,273 days | Two aspirate smear slides, destained Paps | 30%; 50 mm² area | *KIF5B::RET*.K15R12.COSF1232 | Concordant | STGA RNA |
| **Case 32**  75, FFNA lung—NSCLC Stage IV | 28 days | Brigatinib: 36 days  Lorlatinib: 988 days  Active treatment: 1,238 days | Two aspirate smear slides, destained Paps | 25%; 50 mm² area | *EML4::ALK.*E13A20.COSF408.1 | Concordant | STGA RNA |
| **Case 33**  40, FFNA lung—follicular variant papillary thyroid carcinoma Stage IV | 27 days | Radiation (status post remote thyroidectomy): 118 days  Partial remission on active surveillance: 1,232 days | One aspirate smear slide, destained DQ | 40%; 150 mm² area | *CCDC6::RET*.C1R11 | Concordant | MAPP |
|  |  |  | One aspirate smear slide, destained Pap | 40%; 200 mm² area | *CCDC6::RET*.C1R11 | Concordant | MAPP |
| **Case 34**  43, MFNA 4R lymph node—NSCLC Stage IV | 8 days | Carboplatin, pemetrexed, and radiation (*initiated at an outside institution before transfer to MDACC*): –345 days prior to current sample collection  Crizotinib and radiation: 11 days  Lost to follow-up: 92 days | Two aspirate smear slides, destained Pap and DQ | 20%; 100 mm² area | *EZR::ROS1*.E10R34.COSF1267 | Concordant | STGA RNA |
| **Case 35**  71, FFNA station 7 lymph node—NSCLC Stage IV | 35 days | Pembrolizumab (*initiated at an outside institution before transfer to MDACC)*: –12 days prior to current sample collection  Alectinib: 106 days  Deceased: 117 days | Two aspirate smear slides, destained Pap and DQ | 20%; 25 mm² | *EML4::ALK*.E18A20.COSF487.1 | Concordant | STGA RNA |
|  |  |  | One aspirate smear, destained Pap | 20%; 100 mm² | *EML4::ALK*.E18A20.COSF487.1 | Concordant | STGA RNA |
| **Case 36**  39, MFNA station 7 lymph node—NSCLC Stage IV | 13 days | Lorlatinib: 15 days  Carboplatin, pemetrexed: 831 days  Deceased: 842 days | FFPE cell block, one 10 μm section | 40%; 100 mm² area | *EML4::ALK*.E13A20.COSF408.1 | Concordant | STGA RNA |
| **Case 37**  77, FFNA station 7 lymph node—NSCLC Stage IV | 21 days | Carboplatin: 11 days  Capmatinib: 77 days  Elzovantinib clinical trial: 221 days  Deceased: 453 days | One aspirate smear slide, destained DQ | 40%; 300 mm² area | *MET::MET.*M13M15 | Concordant | STGA RNA |
| **Case 38**  36, MFNA station 7 lymph node—NSCLC Stage IV | 16 days | Lorlatinib: 16 days  Carboplatin and pemetrexed: 831 days  Deceased: 842 days | Two aspirate smear slides, destained Pap and DQ | 30%; 200 mm² area | *EML4::ALK*.E13A20 | Concordant | STGA RNA |
| **Case 39**  60, MFNA station 7 lymph node—NSCLC Stage IV | 35 days | Repotrectinib and radiation: 37 days  Deceased: 102 days | Two aspirate smear slides, destained Pap and DQ | 30%; 100 mm² | *SDC4::ROS1.*S2R32.COSF1265 | Concordant | STGA RNA |
|  |  |  | FFPE cell block, one 10 μm section | 30%; 150 mm² | *SDC4::ROS1.*S2R32.COSF1265 | Concordant | STGA RNA |
| **Case 40**  27, MFNA submandibular gland—mammary analogue secretory carcinoma Stage III | 18 days | Surgery and subsequent radiation: 412 days  In remission and on surveillance: 797 days | FFPE cell block, one 10 μm section | 50%; 150 mm² area | *ETV6::NTRK3*.E5N14 | Concordant | STGA RNA |
| **Case 41**  58, M  FNA  station 7 lymph node—NSCLC Stage IV | 23 days | Carboplatin and pemetrexed (initiated before transfer to MDACC): –300 days prior to current sample collection  Alectinib: 93 days  Deceased: 240 days | Two aspirate slides, destained Pap and DQ | 20%; 25 mm² area | *EML4::ALK*.E19A20 | Discordant—fusion not detected; no fusion-specific or expression-imbalance signal | STGA RNA |
| **Case 42**  43, F  FNA station 7 lymph node—NSCLC Stage IV | 20 days | Carboplatin and pemetrexed (initiated before transfer to MDACC): –120 days prior to current sample collection  No targeted therapy administered  Deceased: 232 days | Two aspirate slides, destained DQ | 20%; 50 mm² area | *EML4::NTRK3*.E2N14 | Discordant—equivocal for *NTRK3* | STGA RNA |
